# Prevalence of depression among the elderly (60 years and above) population in India, 1997–2016: a systematic review and meta-analysis

**DOI:** 10.1186/s12889-019-7136-z

**Published:** 2019-06-27

**Authors:** Manju Pilania, Vikas Yadav, Mohan Bairwa, Priyamadhaba Behera, Shiv Dutt Gupta, Hitesh Khurana, Viswanathan Mohan, Girish Baniya, S. Poongothai

**Affiliations:** 1 RUHS College of Medical Sciences, Jaipur, India; 2 Atal Bihari Vajpayee Government Medical College, Vidisha, India; 3 IIHMR University, Jaipur, India; 4 Department of Community Medicine and Family Medicine, AIIMS, Bhubaneshwar, India; 5 Pt B D Sharma Postgraduate Institute of Medical Sciences, Rohtak, India; 6 Madras Diabetes Research Foundation, Chennai, India; 7 SP Medical College, Bikaner, India

**Keywords:** Prevalence, Depression, India, Elderly, Systematic review, Meta-analysis

## Abstract

**Background:**

There is lack of information on the magnitude of depression among elderly population in India. This systematic review and meta-analysis aimed to estimate the prevalence of depression among elderly population in India.

**Methods:**

PubMed, Scopus, Web of Science, Embase, PsycINFO, IndMed, and Google Scholar were searched to identify articles reported community-based prevalence of depression among elderly population using screening tools. This study included the articles published during the years 1997 to 2016. Studies conducted in the special population groups, hospitals, reported only a subcategory of depression, and not specified the screening tool were excluded. Data were extracted from published reports and any missing information was requested from authors. Estimates were pooled using random-effects meta-analyses. Subgroup and sensitivity analysis were performed. The publication bias was evaluated by using Egger’s test and visual inspection of the symmetry in funnel plots.

**Results:**

Fifty-one studies from 16 States of India were included as 56 datasets, which estimated the prevalence of depression among Indian elderly population as 34.4% (95% CI: 29.3–39.7). In sub-group analysis, the pooled prevalence was higher among females, rural populations, and in the eastern part of the country. Studies using non-probability sampling, and GDS and CES-D screening tool showed higher prevalence. Exclusion of the studies with sample size less than 100 and low-quality studies (score < 5/8) had no effect on the estimate of the prevalence. The studies that excluded dementia before assessment of depression had lower prevalence.

**Conclusion:**

About one third elderly population of India suffered from depression with female preponderance. The estimates varied with type of study tool, geographic region, sampling methods, and presence of dementia. The pooled estimate should be interpreted with caution as the studies included in this review had varied methodological approach and screening tools.

**Electronic supplementary material:**

The online version of this article (10.1186/s12889-019-7136-z) contains supplementary material, which is available to authorized users.

## Background

Depression is a major mental health problem, which is yet to be recognised as an important public health challenge. About 322 million people affected with depression worldwide [1]. Depression is the single largest contributor to global disability (7.5%, 2015) and a major contributor to suicides (~ 800,000 annually) [2]. In India, elderly persons (60 years and above) constitute 8.6% of the total population (India Census 2011), which is projected to reach 19% by 2050 [3]. Thus, depression among elderly population is likely to be a major cause of disease burden in the future.

Depression is one of the most common illnesses in the elderly population. Among elderly people, chronic diseases, restricted mobility, bereavement, elderly abuse, isolation, and loss of income are major risk factors for depression, in addition to common risk factors in all age groups [4]. Depression in the elderly persons may have a varied presentation and may be difficult to diagnose [5]. It has devastating consequences and contributes significantly to misery in this phase of life [6]. It is associated with increased risk of morbidity, decreased physical, cognitive and social functioning, and greater self-neglect [4, 7]. Depression not only decreases the quality of life but also influence prognosis of other chronic diseases that further aggravates disability [8]. Consequently, elderly persons with depression have significantly higher suicidal and non-suicidal mortality [9]. Early identification and management of depression can improve quality of life. However, healthcare systems in low and middle income countries like India are not resilient enough to deal with mental health problems including depressive disorders [10].

There are no systematically conducted and nationally representative studies in India like China Biobank Study [11], which provide data on disease magnitude to address the nation’s need for developing the policies and strengthening programs [12]. Current epidemiological studies do not provide a solution to this challenge as there is a wide variation in estimated prevalence ranging from 6 to 80% depending on various factors like population studied, screening tool used etc. [13, 14]. Hence, we conducted this systematic review and meta-analysis with aim to estimate the prevalence of depression among elderly population in India.

## Methods

### Protocol and registration

This systematic review is reported in accordance with the PRISMA checklist [15], and is registered in the PROSPERO database (International Prospective Register of Systematic Reviews) (CRD42014014691) [16].

### Information sources and search strategy

We searched PubMed, Scopus, Web of Science, Embase, PsycINFO, and IndMed for articles published during the years 1997 to 2016. One hundred pages of Google scholar were reviewed to enrich and supplement the search results [17, 18]. No language restriction was imposed in the searches. The cross-references of the identified studies were explored for additional studies. Keywords were identified with discussion among authors, and search query was developed for respective databases (Additional file 1).

### Eligibility criteria

We included the studies reporting prevalence of depression using screening tools.

#### Inclusion criteria

1). Community-based studies; 2). Participant’s age - 60 years and above; 3). Type of studies - cross-sectional studies, and cohort studies; 4). Studies published during the years 1997 to 2016 to provide depression prevalence from previous two decades.

#### Exclusion criteria

1). Studies conducted in the special population groups such as chronic disease patients; 2). Studies conducted in special settings such as old age homes and hospitals; 3). Studies that reported only subcategory of depression; 4). Studies which have not reported the screening tool.

### Data extraction (selection and coding)

Two authors (MP, PB) individually involved in the extraction of relevant studies from the databases. All the eligible studies were screened; in case of duplication of information, we chose the latest article with maximum information. After selection of eligible studies, study characteristics and relevant data, namely author (year of publication), study location and setting (State: rural and urban), study design, sampling method, sample size, response rate (%), screening tool, prevalence, and screening for dementia were extracted. We contacted authors through email for additional data whenever required. Discrepancies in data were resolved either by consensus or seeking additional information from the author(s) of the study. In case of disagreement between two reviewers (MP and PB), arbitration was done by other authors (MB and VY).

### Risk of Bias assessment

We used Adapted New Castle Ottawa Scale for cross-sectional studies to assess the quality in terms of representativeness, sample size, comparability, non-response, ascertainment of outcome and statistical analysis [19, 20] and quality scores were assigned to each study (Additional file 2). Sensitivity analysis was done to remove the influence of low-quality studies, small studies, and presence of dementia.

### Strategy for data synthesis

The effect size of interest for this study was the prevalence of depression among elderly population. Pooled estimates were calculated separately for males, females, and combined population. Freeman-Tukey Double arcsine transformation of proportions are implemented to calculate all pooled estimates, as it is preferred method for calculating effect size for proportions [21]. Stata 13 was used to calculate of pooled effects, subgroup analysis, publication bias analysis, forest plot and sensitivity analysis (StataCorp. 2013. Stata Statistical Software: Release 13. College Station, TX: StataCorp LP.). Meta-regression analysis was done in R software using Meta and Metafor packages [22, 23]. Heterogeneity between studies was examined using the Isquared statistic and Cochran’s Q test. Due to significant heterogeneity between the studies (I^2^ = 98.5% and Cochran’s Q = 3574.8, df = 55, *p* < 0.001), we used random effects models for analysis [24, 25]. All pooled estimates were calculated using DerSimonian and Laird method of random effects models and reported as a proportion with 95% confidence interval [26, 27]. We assessed the publication bias by visual inspection of funnel plots and Eggers test. Funnel plot was made between transformed proportions and standard error of transformed proportions. Egger’s method for detecting publication bias was originally described for effect size based on odds ratio but this test can be applied to effect size calculated by any method. According to this method, asymmetry in funnel plots is tested by carrying out a simple linear regression of *yi* (the effect size in study *i* divided by its standard error) on *xi* (the inverse of the standard error) and testing whether the intercept significantly differs (at *p* < 0.1) from zero. Statistical significance was set at *p*-value < 0.05. Subgroup analysis of combined estimate of the prevalence was done for residence, region, screening tools, time-period (1997–2006 and 2007–2016), and sampling methods.

India is a federal country comprising of 29 States and 7 Union Territories. It has been divided into four regions namely, the North and Central region included Jammu and Kashmir, Himachal Pradesh, Haryana, Delhi, Uttarakhand, Uttar Pradesh, Madhya Pradesh, and Chhattisgarh; the South region included Tamil Nadu, Andhra Pradesh, Karnataka, and Kerala; the East region included West Bengal, Bihar, Jharkhand, Odisha, Assam, and other north eastern states; and the West region included Gujarat, Goa, and Maharashtra. Based on socioeconomic status, the States have been divided into Empowered Action Group (EAG) states & non-EAG states in the country by Government of India. The eight socioeconomically backward States, Bihar, Jharkhand, Madhya Pradesh, Chhattisgarh, Odisha, Rajasthan, Uttarakhand and Uttar Pradesh are classified under the EAG states [28].

## Results

**Fig. 1:**
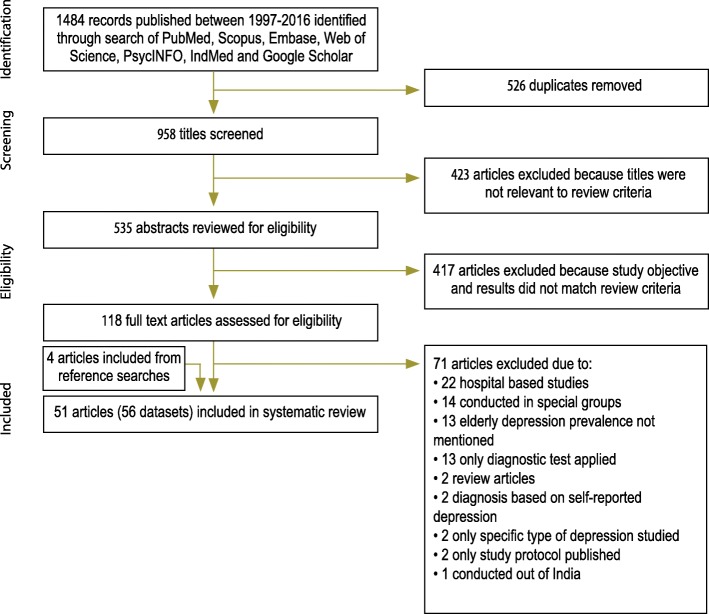
PRISMA flowchart of selection of studies

In this systematic review, 51 studies fulfilled eligibility criteria (Fig. 1). We have planned a priori to estimate the prevalence of rural and urban separately. Of the 51 studies, 6 studies were conducted in both rural and urban community. We divided these 5 articles into two parts each, rural and urban; and 1 stood undivided due to lack of data. Hence, we analysed these 51 studies as 56 datasets [46 original datasets + 10 datasets from 5 studies (each had one urban and one rural dataset)]. In total, there were 22,005 study subjects, the smallest sample size being 41, and largest 2186. The main characteristics of the selected studies have been summarized in Table 1.

**Table 1:** Characteristics of the studies selected in the systematic review of the prevalence of depression in elderly population, India, 1997–2016

S.No	Author, Year of Publication (study number)	State/ Study Setting	Sampling technique†	Age (yrs)	Screening tool	Dementia patients excluded	Combined Prevalence in % ‡	Prevalence in males,% §	Prevalence in females,%¶	Quality Score
1	Abhishekh HA et al., 2013	Karnataka/ Rural	US	≥ 60	HDRS	No	14.3 (10/70)	12.1 (4/33)	16.2 (6/37)	5
2	Ahmed MS et al, 2016	Karnataka/ Urban	SyRS	≥ 60	GDS – 15	No	36.7 (312/850)	32.3 (132/409)	40.8 (180/441)	7
3	Arumugam B et al, 2013 (A)	Tamil Nadu/Rural	US	≥ 60	GDS – 30	No	79.5 (66/83)	66.7 (18/27)	85.7 (48/56)	4
4	Arumugam B et al, 2013 (B)	Tamil Nadu/Urban	US	≥ 60	GDS – 30	No	80 (72/90)	63.3 (19/30)	88.3 (53/60)	4
5	Arvind P et al., 2004	Kerala/ Rural	SyRS	≥ 60	GDS – 15	No	24.7 (64/259)	18.5 (22/119)	30.0 (42/140)	8
6	Barua A et al., 2010	Karnataka/Rural	SiRS	≥ 60	MDIPC v2.2	Yes	21.7 (132/609)	19.9 (43/216)	22.6 (89/393)	8
7	BayapareddyPM et al.,2012	Tamil Nadu/ Rural	CRS	≥ 60	GDS – 15	Yes	47 (376/800)	37.5 (150/400)	56.5 (226/400)	7
8	Behera P et al., 2016	Haryana/ Rural	SiRS	≥ 60	GDS – 30	Yes	27.3 (108/395)	23.3 (40/172)	30.5 (68/223)	8
9	Bodhare TN et al, 2013	Andhra Pradesh/ Rural	US	≥ 60	PHQ 9	No	44.7 (85/190)	–	–	3
10	Dasgupta A et al, 2013	West Bengal/Rural	CS	≥ 60	GDS – 15	No	58.8 (50/85)	48.4 (15/31)	64.8 (35/54)	7
11	Dasgupta A et al, 2014	West Bengal/Urban	StRS	≥ 60	GDS – 15	No	46.9 (61/130)	36.1 (22/61)	56.5 (39/69)	7
12	Deshpande SS et al., 2011	Maharashtra/ Rural	SyRS	≥ 65	GDS – 15	No	41.1 (74/180)	40.2 (37/92)	42.0 (37/88)	6
13	Dhar G et al., 2013	West Bengal/Urban	SyRS	≥ 60	GDS – 15	No	59.8 (122/204)	–	–	5
14	Dhuria M et al., 2014	Delhi/ Urban	Not known	≥ 60	GDS – 15	No	45.6 (114/250)	–	–	2
15	D’souza L et al., 2015	Karnataka/Urban	Not known	≥ 60	GDS – 15	No	51.9 (109/210)	33 (35/106)	71.2 (74/104)	4
16	Dumbray SS et al, 2014	Maharashtra/ Urban	CS	≥ 60	GDS – 15	No	30 (30/100)	–	–	5
17	Ganguli M et al., 1999	Haryana/ Rural	Not known	> 60	GDS – 30	Yes	46.4 (646/1391)	40.4 (294/727)	53 (352/664)	7
18	Goel PK et al., 2014	Uttar Pradesh/Urban	SyRS	≥ 60	GDS – 30	No	9.4 (38/403)	9.7 (20/207)	9.2 (18/196)	7
19	Goyal A et al., 2014 (A)	Punjab/ Rural	Not known	≥ 60	GDS – 30	No	74.6 (44/59)	–	–	5
20	Goyal A et al., 2014 (B)	Punjab/Urban	Not known	≥ 60	GDS – 30	No	80.5 (33/41)	–	–	5
21	Gupta A et al., 2015	Uttar Pradesh/Urban	MRS	≥ 60	GDS – 30	Yes	15.6 (22/141)	11 (11/100)	26.8 (11/41)	4
22	Gupta SK et al., 2012	Madhya Pradesh/ Urban	SyRS	≥ 60	GDS – 15	No	9.6 (20/208)	12.1 (11/91)	7.7 (9/117)	4
23	Ishikawa M et al, 2016	Jammu and Kashmir/ Rural	PS	≥ 60	PHQ-2	No	7.9 (9/114)	–	–	4
24	Jain RK et al., 2007	Maharashtra/ Urban	LQS	≥ 60	GDS – 15	No	45.9 (90/196)	38 (38/100)	54.2 (52/96)	7
25	Jariwala V et al.,2010	Gujrat/Urban	CS	≥ 60	BDI (G)	No	35.7 (25/70)	–	–	5
26	Jonas Jost B et al, 2014	Maharashtra/ Rural	CS	≥ 60	CES-D	No	58.5 (802/1370)	48.1 (311/647)	67.9 (491/723)	8
27	Kamble SV et al, 2009	Maharashtra/ Rural	SyRS	≥ 60	Goldberg & Bridges scale	No	31.4 (155/494)	24.6 (57/232)	37.4 (98/262)	8
28	Kumar S et al., 2013	Andhra Pradesh/ Rural	CRS	≥ 60	GDS – 15	Yes	47 (188/400)	37.5 (75/200)	56.5 (113/200)	7
29	Mathias K et al. 2015	Uttarakhand/ Unclassified	2 s CRS	≥ 60	PHQ 9	No	5.5 (6/109)	–	–	7
30	Maulik S et al., 2012	West Bengal/Rural	CRS	≥ 60	GDS- 15 (Bengali)	No	53.7 (44/82)	33.3 (9/27)	63.6 (35/55)	7
31	Nair SS et al., 2013	Karnataka/ Urban	SiRS	≥ 60	GDS – 15	No	32.4 (59/182)	32.0 (24/75)	32.7 (35/107)	3
32	Nandi PS et al., 1997	West Bengal/Rural	US	≥ 60	WHO TRS	Yes	55.2 (101/183)	37.6 (32/85)	70.4 (69/98)	4
33	Patil SD et al., 2015	Karnataka/Rural	SyRS	≥ 60	GDS – 15	No	29.4 (114/388)	28 (37/132)	30.1 (77/256)	7
34	Payghan BS et al, 2013	Karnataka/Urban	StRS	≥ 60	GDS – 15	No	41.7 (90/216)	38.5 (40/104)	44.6 (50/112)	7
35	Pilania M et al., 2016	Haryana/Rural	2 s CRS	≥ 60	GDS – 30	No	14.4 (72/500)	8.7 (20/231)	19.3 (52/269)	7
36	Pongiya UD et al, 2011	Tamil Nadu/ Rural	Not known	≥ 60	CES-D	No	22 (20/91)	28.3 (13/46)	15.6 (7/45)	3
37	Poongothai S et al, 2009	Tamil Nadu/ Urban	MRS	≥ 60	PHQ 12	Yes	28.5 (622/2186)	25.9(296/1142)	31.2 (326/1044)	8
38	Pracheth R et al., 2013	Karnataka/ Urban	SyRS	≥ 60	GDS – 30	No	29.4 (64/218)	25.9 (21/81)	31.4 (43/137)	7
39	Radhakrishnan S et al., 2013	Tamil Nadu/ Rural	SiRS	≥ 60	GDS – 30	No	58.8 (235/400)	45.2 (76/168)	68.5 (159/232)	7
40	Raul A et al., 2013	Maharashtra/ Urban	Not known	≥ 60	MDIPC v2.2	No	21.3 (46/216)	–	–	–
41	Saikia AM et al., 2016	Assam/Urban	CRS	≥ 60	GDS – 15	Yes	17.3 (69/400)	14.5 (27/186)	19.6 (42/214)	7
42	Sandhya GI et al., 2010	Kerala/ Rural	StRS	≥ 60	GDS – 15	No	25.4 (65/256)	29.1 (30/103)	22.9 (35/153)	7
43	Sanjay TV et al., 2014	Karnataka/Urban	SiRS	≥ 60	GDS – 15 (Kannada)	No	36 (36/100)	29.5 (13/44)	41.1 (23/56)	7
44	Santosh A et al., 2014	Karnataka/Urban	SyRS	≥ 60	GDS	No	33.3 (50/150)	31.1 (14/45)	34.3 (36/105)	7
45	Seby K et al., 2011	Maharashtra/ Urban	US	≥ 65	GDS – 15	Yes	19.3 (39/202)	–	–	4
46	Sengupta P et al., 2015 (A)	Punjab/ Rural	US	≥ 60	GDS – 15	Yes	7.3 (91/1248)	5.7 (33/579)	8.7 (58/669)	8
47	Sengupta P et al., 2015 (B)	Punjab/Urban	US	≥ 60	GDS – 15	Yes	10.1 (180/1790)	7.5 (60/805)	12.2 (120/985)	8
48	Sharma K et al., 2016 (A)	Himachal Pradesh/ Rural	2 s CRS	≥ 60	HDRS	No	7.3 (29/400)	–	–	7
49	Sharma K et al., 2016 (B)	Himachal Pradesh/ Urban	2 s CRS	≥ 60	HDRS	No	11.8 (47/400)	–	–	7
50	Sinha SP et al., 2013	Tamil Nadu/ Rural	US	≥ 60	GDS – 15	Yes	42.7 (44/103)	29.3 (17/58)	60 (27/45)	5
51	Suganathan S et al, 2016	Tamilnadu/Rural	CRS	≥ 60	GDS	No	70.4 (317/450)	56.8 (100/176)	79.2 (217/274)	7
52	Sundru M et al., 2013 (A)	Andhra Pradesh/ Rural	SiRS	≥ 60	GDS – 15	No	36 (216/600)	–	–	6
53	Sundru M et al., 2013 (B)	Andhra Pradesh/ Urban	SiRS	≥ 60	GDS – 15	No	27.3 (164/600)	–	–	6
54	Swarnalatha N et al., 2013	Andhra Pradesh/ Rural	SiRS	≥ 60	GDS – 15	No	47 (188/400)	37.5 (75/200)	56.5 (113/200)	7
55	Thirthahalli C et al, 2014	Karnataka/Urban	StRS	≥ 60	CES-D	Yes	37.8 (179/473)	28.8 (40/139)	41.6(139/334)	8
56	Yadav SP et al., 2013	Maharashtra/ Urban	SyRS	≥ 60	GDS – 15	No	15.9 (43/270)	14 (18/129)	17.7 (25/141)	6

†US- Universal Sampling (all eligible participants selected); SyRS – Systematic Random Sampling; SiRS –Simple Random Sample; CRS – Cluster Random Sampling; StRS – Stratified Random Sampling; CS – Convenience Sampling; PS – Purposive Sampling; LQS – Lots Quality Sampling; MRS – Multistage Random Sampling; 2 s CRS – Two stage cluster random sampling

‡No. of positive patients/ Total participants; § No. of positive males / Total males; ¶ No. of positive females / Total females

Most of the studies [29] were published in recent 5 years from 2012 to 2016, followed by 9 during 2007 to 2011, and only 3 from 1997 to 2006. Almost half (*n* = 26) of the studies were conducted in South India, followed by North (*n* = 14), West (*n* = 9), East (*n* = 5), North-East and Central (n = 1, each). Only 4 studies were conducted in EAG states and 1 in Assam. Most of the studies (*n* = 43) used probability sampling methods, and 6 studies used non-probability sampling methods. Seven studies did not mention the type of sampling method adopted.

Geriatric Depression Scale (GDS) was most commonly (*n* = 41) used screening tool [13, 29–65] followed by Public Health Questionnaire (PHQ) 4 studies [14, 66–68], Center for Epidemiologic Studies Depression Scale (CES-D) in 3 studies, [69–71]), Hamilton Depression Rating Scale (HDRS) in 3 studies [72, 73], Mastering Depression in Primary Care Version 2.2 (MDIPCv2.2) in 2 studies [74, 75], and Beck Depression Inventory (BDI) [76], Goldberg and Bridges Scale [77], and definition based on WHO Technical Report Series 1960 [78] each in 1 study. Two studies provided prevalence in persons aged 65 years and above only. Only 14 studies excluded elderly having dementia before applying the screening tool for depression. Only 41 studies provided the prevalence of depression in males and females, separately.

### Prevalence of depression in elderly

**Fig. 2:**
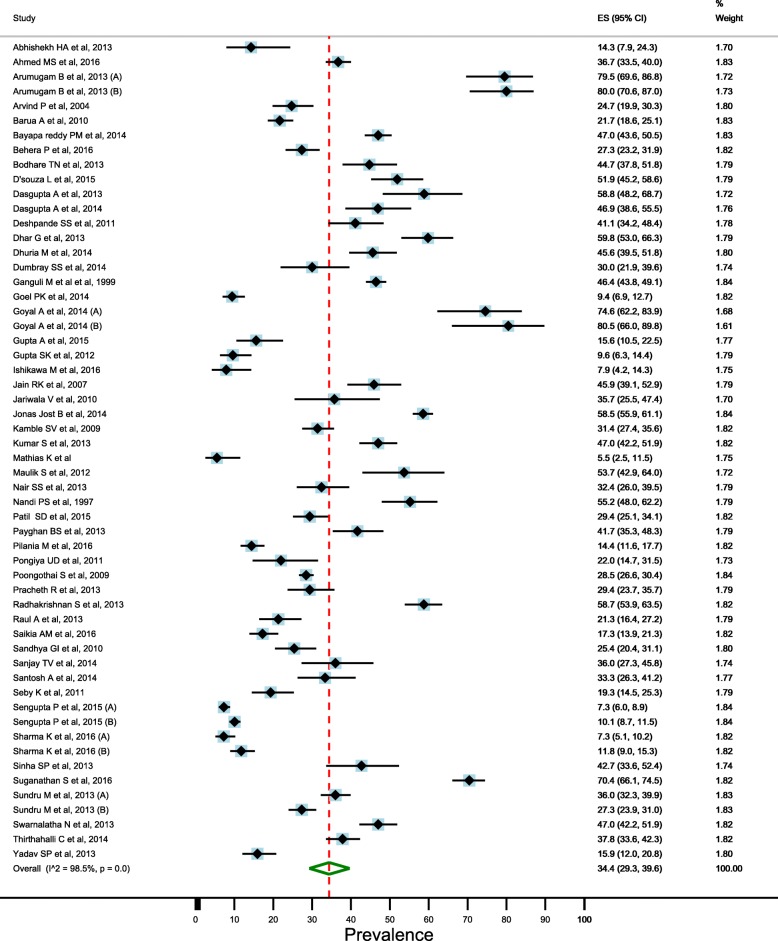
Estimated prevalence of depression among elderly persons in India pooling included studies, 1997–2016

**Fig. 3:**
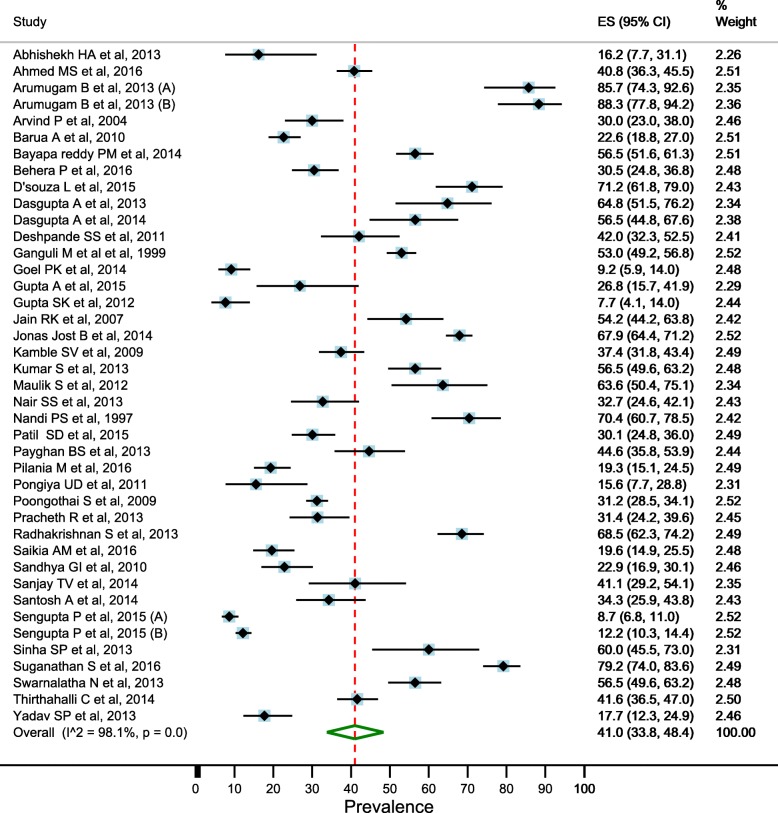
Estimated prevalence of depression among female elderly persons in India pooling included studies, 1997–2016

**Fig. 4:**
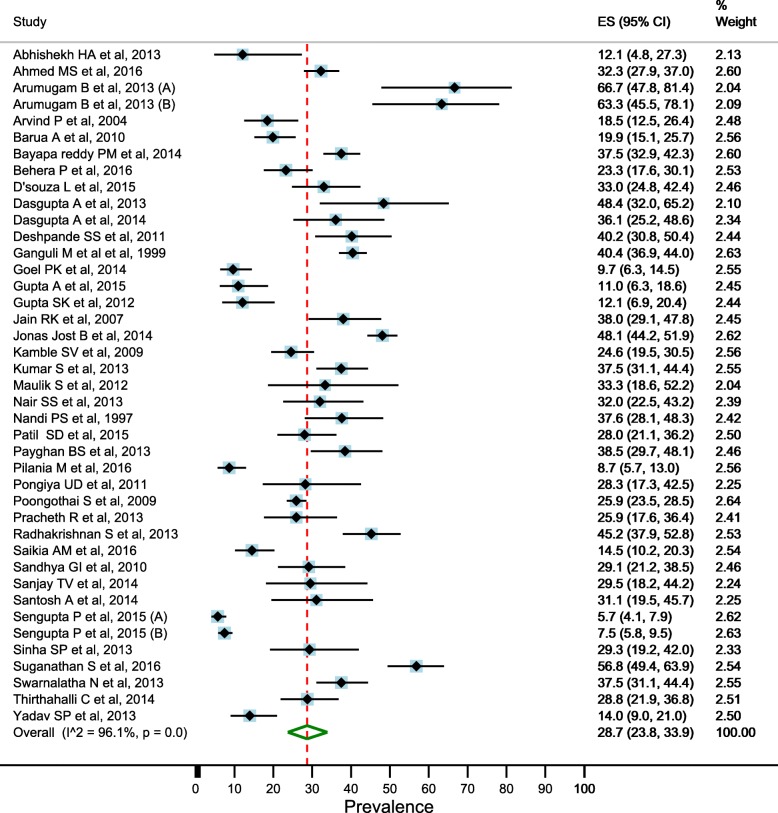
Estimated prevalence of depression among male elderly persons in India pooling included studies, 1997–2016

There was significant heterogeneity between the studies (I^2^ = 98.5% and Cochran’s Q = 3574.8, df = 55, *p* < 0.001), therefore, we used random effects models for estimating the prevalence of depression in elderly. Overall pooled estimate (random effects models) of the prevalence of depression in the elderly was 34.4% (95% CI: 29.3–39.7) (Fig. 2). Pooled estimate of the prevalence was higher in the females than males (41.0%; 95% CI: 33.8–48.4 vs. 28.7%; 95% CI: 23.8–33.9) (Figs. 3 and 4).

### Subgroup analysis

Subgroup analysis is presented in Table 2. Studies from rural areas showed slightly higher prevalence of depression (37.8%; 95% CI: 29.9–45.9) than urban areas (32.1%; 95% CI: 26.1–38.5), however, this difference was not significant (Additional file 3: Figure S1).

**Table 2:** Prevalence of depression in the elderlypopulation using random effects model by subgroup and sensitivity analyses

Category		No. of studies	Pooled prevalence (95% CI)	Cumulative Positives/cumulative sample size	p-value in between group comparison
All studies	Overall	56	34.4 (29.3–39.6)	7087/22005	
Subgroup					
Year of publication	2007–2016	53	34 (28.7–39.5)	6276/20172	0.3525
	2006 and before	3	41.7 (26.8–57.5)	811/1833	
Setting	Rural	28	37.8 (29.9–45.9)	4345/11600	0.2778
	Urban	27	32.1 (26.1–38.5)	2736/10296	
Region	South	26	39.8 (34.5–45.3)	3877/10374	0.0073
	North and Central	15	21.6 (13.3–31.3)	1459/7449	
	East including North-east	6	47.9 (30.1–66.1)	447/1084	
	West	9	32.7 (21.1–45.5)	1304/3098	
State	Andhra Pradesh	5	40.1 (32–48.5)	841/2190	< 0.001
	Assam	1	17.3 (13.9–21.3)	69/400	
	Delhi	1	45.6 (39.5–51.8)	114/250	
	Gujrat	1	35.7 (25.5–47.4)	25/70	
	Haryana	3	28.6 (10.8–50.7)	826/2286	
	Himachal Pradesh	2	9.4 (7.4–11.5)	76/800	
	Jammu and Kashmir	1	7.9 (4.2–14.3)	9/114	
	Karnataka	11	33.1 (27.8–38.5)	1155/3466	
	Kerala	2	25.0 (21.4–28.9)	129/515	
	Madhya Pradesh	1	9.6 (6.3–14.4)	20/208	
	Maharashtra	8	32.3 (20–46.1)	1279/3028	
	Punjab	4	37.4 (20.1–56.6)	348/3138	
	Tamil Nadu	8	53.7 (38.9–68.2)	1752/4203	
	Uttar Pradesh	2	10.9 (8.3–13.6)	60/544	
	Uttarakhand	1	5.5 (2.5–11.5)	6/109	
	West Bengal	5	55.1 (50.5–59.7)	378/684	
EAG states	EAG and Assam	5	11.3 (7.6–15.8)	155/1261	< 0.001
	Non-EAG states†	25	34.3 (25.4–43.8)	3055/10370	
	South Indian states	26	39.8 (34.5–45.3)	3877/10374	
Sampling methods	Probability	43	31.8 (26.4–37.4)	5069/17812	0.0475
	Non-probability	6	38.4 (22.2–55.9)	1006/1935	
	Not known	7	47.7 (36.1–59.4)	1012/2258	
Instrument	CES-D	3	39.5 (21.7–58.9)	1001/1934	< 0.001
	GDS	41	37.9 (31.5–44.5)	4819/15030	
	HDRS	3	10.2 (6.5–14.6)	86/870	
	PHQ	4	19.7 (7.5–35.7)	722/2599	
	Others‡	5	32.3 (21.8–43.8)	459/1572	
Type of instrument	GDS	41	37.9 (31.5–44.5)	4819/15030	0.0291
	Others than GDS	15	25.4 (17.1–34.6)	2268/6975	

†Non-EAG states excluding South Indian states; ‡ “Others” in instruments included MDIPC v2.2, Goldberg and Bridges Scale, and BDI (G)

The estimated pooled prevalence among the studies that used probability sampling was relatively lower (31.8%; 95% CI: 26.4–37.4) compared to those studies used non-probability sampling (38.4%; 95% CI: 22.2–55.9). In the studies with unknown sampling methods, the prevalence was relatively higher (47.7%; 95% CI: 36.1–59.5) (Additional file 3: Figure S2).

We did subgroup analysis as GDS vs. non-GDS (all other than GDS) and found that pooled estimate was higher among studies used GDS tool (37.9%; 31.5–44.5 vs. 25.4%; 17.1–34.6) (Additional file 3: Figure S3). Pooled prevalence estimates for CES-D, GDS, PHQ, and HDRS were 39.5% (95% CI: 21.7–58.9), 37.9% (95% CI: 31.5–44.5), 19.7% (95% CI: 7.5–35.7), and 10.2% (95% CI: 6.5–14.6), respectively. “Others”, which included MDIPCv2.2, BDI, Goldberg Bridges Scale, and definition based on WHO Technical Report Series 1960 (No. 185) had a pooled prevalence of 32.3% (95% CI: 21.8–43.8) (Additional file 3: Figure S4).

**Fig. 5:**
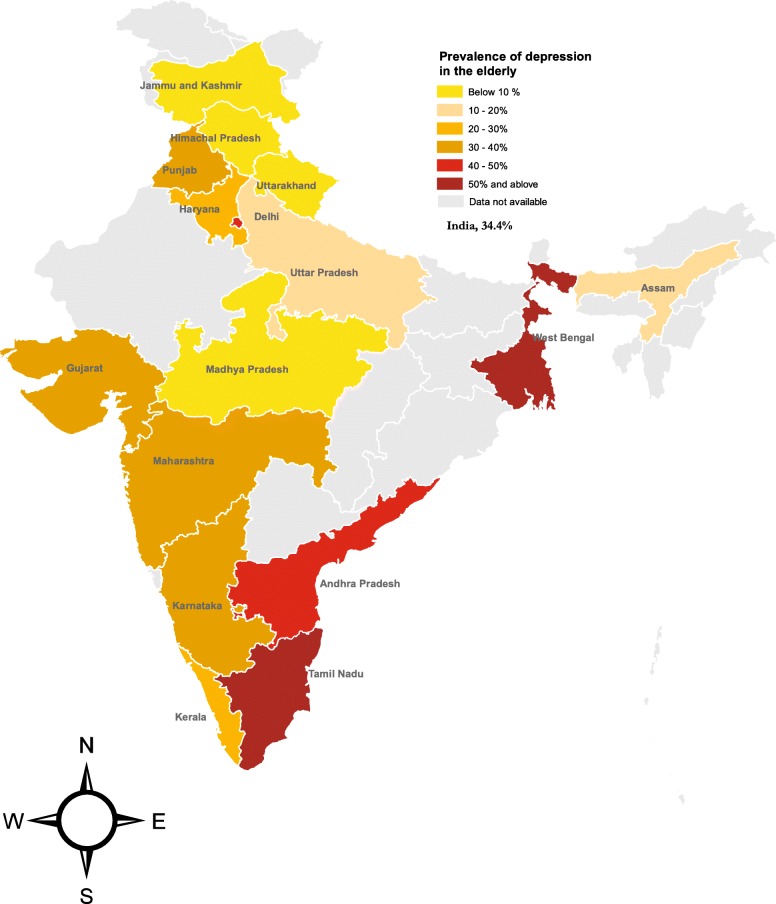
Estimated prevalence of depression among elderly persons in States of India, 1997–2016. Map was created by authors using ArcGIS 10.5 (ESRI, RedLands, USA)

The prevalence in East (including North-East), South, West and North (including Central) zone was 48% (95% CI: 30.1–66.1), 39.8% (95% CI: 34.5–45.3), 32.7% (95% CI: 21.1–45.5), 21.6% (95% CI: 13.3–31.3) respectively (Additional file 3: Figure S5). We divided the states into EAG states, South, and Others (including rest Non-EAG states from East, West, and North India), and found the pooled prevalence of depression as 11.3% (95% CI 7.6–15.8), 39.8% (95% CI 34.5–45.3), and 34.3% (95% CI 25.4–43.8), respectively (Additional file 3: Figure S6). Pooled prevalence in the individual states is also presented in the map of India (Fig. 5).

There was no significant difference between the prevalence from decades 2007–2016 and 1997–2006; the estimated pooled prevalence was 34% (95% CI 28.7–39.5) and 41.7% (95% CI 26.8–57.5), respectively (Additional file 3: Figure S7).

### Quality assessment

Out of 56 studies (56 datasets are considered as 56 studies in our analysis), 55 studies were assessed for quality. One study cannot be assessed for quality because of lack of full text. The quality score for the studies varied from 2 to 8. The median quality score for the studies was 7, Interquartile range = 5,7. There were 9 high quality studies (score 8), 34 medium quality studies (score varies from 5 to 7) and 12 low quality studies (score < 5). The quality score of each study was provided in Table 1.

### Sensitivity analysis

**Fig. 6:**
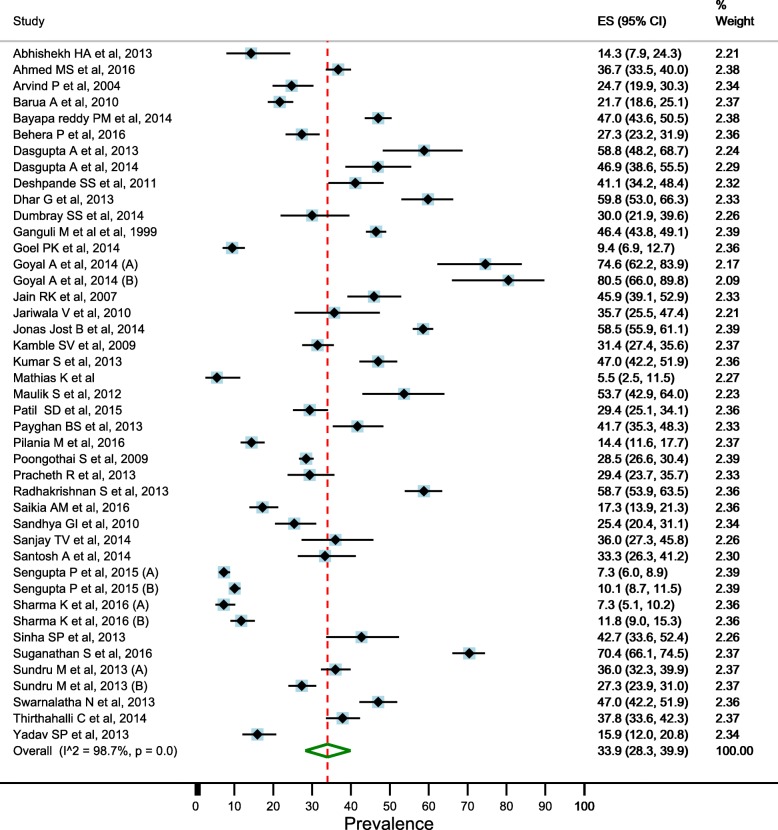
Estimated prevalence of depression among elderly persons in India pooling good quality studies (equal or more than 5) only, 1997–2016 (sensitivity analysis)

We did sensitivity analysis using the random effects model to identify the effect of individual studies on the pooled estimate. No significant changes in the pooled prevalence was found on removal of low-quality studies. Prevalence estimate was 33.9% (95% CI 28.3–39.9, I^2^ = 98.6%, *p* < 0.0001) after omitting the studies with quality score less than 5 (Fig. 6). We found that the pooled prevalence was 34.6% (95% CI 29.3–40, I^2^ = 98.5%) on the removal of 2 studies (with age group 65 years and above) (Additional file 3: Figure S8). The pooled prevalence was lower (30.7%; 95% CI 25.4–36.3, I^2^ = 98.6%) when we excluded studies with the sample size less than 100 (Additional file 3: Figure S9). The estimate was also lower (28.9%; 95% CI 20.3–38.4, I^2^ = 99%) when we omitted the studies which have not screened for dementia (Additional file 3: Figure S10).

### Meta-regression analysis

Mixed effects meta-regression analysis done with study period, residence, geographic region, sampling methods, and screening tool showed that ‘geographic region’ was the only significant covariate that independently and significantly affected the prevalence. The overall model was significant with an r^2^  =  0.50 and *p* value < 0.0001 (Table 3).

**Table 3:** Mixed effects meta-regression analysis – effect of covariates on the prevalence of depression

Covariate	Coefficient	95% CI	SE	Z	P value
Study period (2007–2016)	0.02	−0.17, 0.21	0.096	0.23	0.82
Urban	−0.08	−0.16, 0.006	0.043	−1.82	0.07
Unclassified	−0.11	−0.47, 0.24	0.18	−0.61	0.54
Southern region	− 0.07	− 0.22, 0.07	0.07	−1.04	0.30
North and Central region	−0.31	−0.47, − 0.15	0.08	−3.87	0.0001
Western region	−0.18	−0.35, − 0.01	0.086	−2.11	0.035
Probability sampling	−0.14	−0.30, 0.02	0.08	−1.71	0.087
Unknown sampling methods	0.12	−0.07, 0.31	0.099	1.23	0.22
GDS	0.11	−0.07, 0.30	0.09	1.19	0.23
HDRS	−0.09	−0.35, 0.17	0.13	−0.67	0.51
PHQ	−0.026	−0.27, 0.22	0.13	−0.21	0.83
Others	−0.006	−0.23, 0.22	0.12	−0.05	0.96

Coefficient is for logit of proportion

Dependent variable: prevalence of depression

Reference categories of independent variables: time period 1997–2006, residence - rural, geographic region - east and north-east, sampling methods- non-probability sampling, screening tool - CES-D

### Publication Bias

**Fig. 7:**
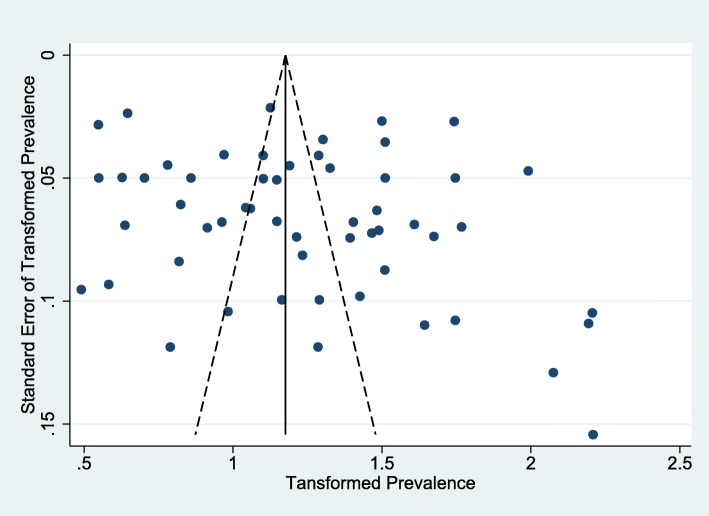
Funnel plot with pseudo 95% confidence limits

The studies had a high degree of heterogeneity (I^2^ = 98.3%). Egger test (no small study effects, intercept = 3.22, t-value = 1.33, *p*-value = 0.189) did not show any evidence of publication bias. The funnel plot (Fig. 7) is of reasonably symmetrical shape which further supports the findings of Egger’s test.

## Discussion

This meta-analysis provided an estimate of the prevalence of depression from 56 community based studies. We found that the pooled prevalence of depression in the elderly population in India was as high as 34.4%. The scope of service provision, improvement is the need of hour specifically designed to equip with the mental health of elderly persons. Sudies from other low and middle income countries also documented high prevalence of depression in the elderly population. The estimated pooled prevalence of depression in China was 22.7% [20] and 23.6% [21] from two recently published meta-analyses. WHO Study on Global Ageing and Adult Health (2007–10) documented higher prevalence of the depression in the Indian elderly population than other low and middle income countries such as China, Ghana, Mexico, Russia, and South Africa [79]. A recently published systematic review of 26 studies among Iranian elderly populations estimated the prevalence of depression (43%) to be higher than India [80]. These differences in the results may be explained by different culture, genetics, and environmental factors or even methodological/ sampling differences. However, taken together, they all support an argument for placing greater importance on the mental health of elderly people, as a part of overall efforts to improve quality of life. In coming years, India will have greater number of elderly people with depression not only due to high prevalence of depression but also increasing share of elderly population. Hence, re-orentation of scarce mental health services and resources including untapped potential of community health workers and new age technology may be useful. In addition, Government of India’s recently lauched ambitious scheme Ayushman Bharat also has a great opportunuty to address mental health needs through health and wellness centres and national health protection scheme.

This systematic review included studies from 1997 to 2016. This period marked the era of broad access to communication technologies like mobile phone and various applications, Internet, E-health and online access to health information [81]. Also during this time, majority of Indian families moved from joint families to nuclear families, and the younger generation migrated to the towns and cities which further weakened the support structures for elderly population, without any signicant improvement in the care and support services for the elderly population [82–84].

Most of the studies on depression among elderly persons published in the last 5 years i.e. from 2012 to 2016. This may be a reflection of increased focus of researchers on problems of elderly persons after adopting aging as an annual theme by WHO in 2012 [85]. In the same year, the World Federation for Mental Health also adopted depression as its theme for World Mental Health day, which further accentuated the awareness for depression in the elderly [86]. The recent increase in publications might also be due to a growth in the number of online research journals [87]. Our study did not find significant difference in the prevalence by time period. Small sample size in the previous decade (1997–2006) may be possible explanation for it. Similarly, the studies which used probability sampling also reported lower prevalence which may be explained by better representativeness and lesser selection bias.

In this systematic review, we idendified studies from 16 states of India. The eastern part of the country had higher prevalence of depression (48%) compared to all other regions. One possible explanation for this finding may be that all the five studies included from the East and North-East zone had used GDS tool which has higher sensitivity [88]. EAG states and Assam lag behind in the demographic transition and have constituted about 46% of India’s population [89]. We found only 5 studies from these states with pooled prevalence of 11.4%. This underscores the need for more research and data on disease burden for effective planning and policymaking in EAG states. Further variable prevalence from different states emphasize on state specific efforts to address this gap, both on the front of research and policy.

In our study, females had higher prevalence of depression which is consistent with global findings and the results of other meta-analyses [90–95]. Diatheses to preponderance of depression in elderly females are vulnerabilities that make them susceptible when stressors occur in their lives. Although, much work was not done on the psychosocial predictors of the gender difference in depression in elderly population, the studies have mostly explored single possible variable such as widowhood/ living alone, poor health, poverty, cognitive decline, caregiving [92, 93, 96].

We found that the prevalence of depression was marginally higher in the rural areas than urban counterparts; however, this was not statistically significant. A systematic review in Chinese rural elderly populations also reported higher prevalence of depression than urban counterparts (29.2% vs. 20.5%) [90]. No specific pattern was observed in other countries [97, 98].

In this meta-analysis, the estimated prevalence of depression in Indian elderly persons varied with different screening criteria. Standardization of the methods and screening tool is essential for assessment of the magnitude of depression among elderly persons in India. The prevalence was higher when CES-D and GDS were used (39.5 and 37.9%, respectively). Since, majority of studies had used the GDS as a screening tool for assessment of depression which is a highly sensitive than others [88]; there is likelihood of overestimation of the prevalence. There was significant heterogeneity in the prevalence between the screening tools, which might be due to different levels of sensitivity and specificity of the screening tools. Another possible explanation may be that most of these screening tools were not validated in the local settings and languages. However, the level of heterogeneity is used to be high in the prevalence studies by nature; standardization of the methods for a uniform assessment of the magnitude of depression or alternatively large cross-sectional studies with standardized assessment tools may also be employed.

On sensitivity analysis, we found that studies with quality score below 5 (of 8) and studies with age group 65 years and above had not affected the pooled estimate. The studies which excluded dementia before assessment of depression and those with large sample size (above 100) reported lower prevalence rates. Exclusion of dementia may limit the number of false positive cases of depression during the assessment. We therefore suggest that the exclusion of dementia is required before screening of depression. We did not find evidence of small studies effect in this review which may have overestimated the effect size.

### Limitations

Most of studies conducted were from South, North and Western region of India and there was no studies from 20 States and Union Territories (UTs) of the total 36 States and UTs in India, although, left out were small States/UTs. Screening tools cannot take the place of a comprehensive clinical interview for confirming a diagnosis of depression; however, it is useful tool for public health programs. Screening provides optimum result when linked with confirmation by psychiatrist, treatment and follow-up. As this meta-analysis included studies using a screening tool, the further meta-analysis on the diagnostic tool will help to estimate the true burden of depression and to determine the need of pharmacological and non-pharmacological interventions.

## Conclusion

This meta-analysis reports that in India, the aggregate prevalence of depression among elderly population was 34.4%, though estimates varied widely throughout the country. Given the varied methodological approaches and screening tools used in the studies included in the review, the appropriateness of calculating pooled prevalence estimates could be questioned. Hence, the pooled prevalence estimate should be interpreted with caution. Despite the limitations, the estimates will guide researchers and planners to measure the burden more appropriately in future. It also emphasizes on the need of standardization of the magnitude of depression to further strengthen the public health measures to address the growing problem.

## Additional files

Additional file 1: Search strategy. (DOC 28 kb)

Additional file 2: Adapted New Castle Ottawa Scale. (DOC 42 kb)

Additional file 3: **Figure S1.** Estimated prevalence of depression among elderly persons in India pooling included studies, 1997–2016 (Rural vs. urban – subgroup analysis). **Figure S2.** Estimated prevalence of depression among elderly persons in India pooling included studies, 1997–2016 (Sampling techniques – subgroup analysis). **Figure S3.** Estimated prevalence of depression among elderly persons in India pooling included studies, 1997–2016 (Study instruments – subgroup analysis). **Figure S4.** Estimated prevalence of depression among elderly persons in India pooling included studies, 1997–2016 (Study instrument for geriatric vs. nongeriatric age groups – subgroup analysis). **Figure S5.** Estimated prevalence of depression among elderly persons in India- pooling included studies: 1997–2016 (EAG vs Non-EAG state of India – subgroup analysis). **Figure S6.** Estimated prevalence of depression among elderly persons in India pooling included studies, 1997–2016 (Geographical regions of India – subgroup analysis). **Figure S7.** Estimated prevalence of depression among elderly persons in India pooling included studies, 1997–2016 (Time period – subgroup analysis). **Figure S8.** Estimated prevalence of depression among elderly persons in India pooling included studies, 1997–2016 (Studies with inclusion age > 60 years only – sensitivity analysis). **Figure S9.** Estimated prevalence of depression among elderly persons in India pooling included studies, 1997–2016 (Excluding studies with sample size < 100 – sensitivity analysis). **Figure S10.** Estimated prevalence of depression among elderly persons in India- pooling included studies 1997–2016 (Studies with dementia exclusion – sensitivity analysis). (PDF 237 kb)

## Data Availability

All data generated or analyzed during this study are included in this published article [and its supplementary information files].

## Abbreviations

BDI: Beck Depression Inventory
CES-D: Center for Epidemiologic Studies Depression Scale
EAG: Empowered Action Group
GDS: Geriatric Depression Scale
HDRS: Hamilton Depression Rating Scale
MDIPCv2.2: Mastering Depression in Primary Care Version 2.2
PHQ: Public Health Questionnaire
PRISMA: Preferred Reporting Items for Systemic Reviews and Meta-analysis
PROSPERO: International Prospective Register of Systematic Reviews
UTs: Union Territories
WHO: World Health Organization
