# Supplementary material for: Prevalence of depression among the elderly (60 years and above) population in India, 1997–2016: a systematic review and meta-analysis
Source: BMC Public Health. 2019 Jun 27;19:832. doi: 10.1186/s12889-019-7136-z (PMC6598256; doi:10.1186/s12889-019-7136-z)
Supplement: Supplementary file 1 — Search strategy. (DOC 28 kb) [file 12889_2019_7136_MOESM1_ESM.doc]

**PubMed (search hits - 286)**

(geriatric OR elder OR old* OR aging) AND (psychiatric OR depressi* OR mental) AND (prevalence OR epidemiology) AND India

Filter applied: Humans; 1997-2016

**Scopus (search hits - 177)**

TITLE-ABS-KEY ( ( geriatric OR elder OR old* OR aging ) AND ( psychiatric* OR depressi* OR mental ) AND ( prevalence OR epidemiology ) AND india ) PUBYEAR > 1996 AND ( EXCLUDE ( SRCTYPE , "b " ) ) AND ( EXCLUDE ( DOCTYPE , "re " ) OR EXCLUDE ( DOCTYPE , "ed " ) ) AND ( EXCLUDE ( PUBYEAR , 2017 ) )

**EMBASE (search hits - 489)**

geriatric OR elder OR old* OR aging AND (psychiatric* OR depressi* OR mental) AND (prevalence OR epidemiology) AND india AND [1997-2016]/py AND [humans]/lim AND ([article]/lim OR [article in press]/lim OR [conference abstract]/lim OR [conference paper]/lim OR [letter]/lim OR [note]/lim OR [short survey]/lim)

**Web of Science (search hits - 331)**

TOPIC: ((geriatric OR elder OR old* OR aging) AND (psychiatric OR depression OR mental) AND (prevalence OR epidemiology) AND India)

Refined by: [excluding] PUBLICATION YEARS: (1993 OR 1995 OR 1996 OR 2017) AND [excluding] DOCUMENT TYPES: (EDITORIAL MATERIAL OR NEWS ITEM OR REVIEW)

Timespan: All years. Indexes: SCI-EXPANDED, SSCI, A&HCI, CPCI-S, CPCI-SSH, BKCI-S, BKCI-SSH, ESCI, CCR-EXPANDED, IC.

**PsycINFO** **(search hits - 245)**

(geriatric OR elder OR old* OR aging) AND (psychiatric OR depressi* OR mental) AND (prevalence OR epidemiology) AND India

**Google Scholar (33)**

(geriatric OR elder OR old* OR aging) AND (psychiatric OR depression OR mental) AND (prevalence OR epidemiology) AND India

**IndMed (search hits - 24)**(geriatric OR elder OR old OR aging) AND (psychiatric OR depression OR mental OR depressive) AND (prevalence OR epidemiology) AND India

After articles retrieval we removed additional 101 articles that were published before the year 1997 or after 2016.
