# Supplementary material for: Prevalence of depression among the elderly (60 years and above) population in India, 1997–2016: a systematic review and meta-analysis
Source: BMC Public Health. 2019 Jun 27;19:832. doi: 10.1186/s12889-019-7136-z (PMC6598256; doi:10.1186/s12889-019-7136-z)
Supplement: Supplementary file 2 — Adapted New Castle Ottawa Scale. (DOC 42 kb) [file 12889_2019_7136_MOESM2_ESM.doc]

| **Adapted New Castle Ottawa Scale** | |
| --- | --- |
| **1** | ***Representativeness of the sample* (Maximum 1 star).** |
|  | a) Truly representative of the average in the target population. * (all subjects or random sampling) |
|  | b) Somewhat representative of the average in the target population. * (non-random sampling) |
|  | c) No description of the derivation of the group |
| **2** | ***Sample size* (Maximum 1 star).** |
|  | a) Justified and satisfactory.* [Large multi-centric studies will be classified as justified and satisfactory sample size.] |
|  | b) Not justified. |
|  | c) No description of the sample size calculation. |
| **3** | ***Non-respondents* (Maximum 1 star).** |
|  | a) Comparability between characteristics of respondents and non-respondents is established, **OR**  the response rate is satisfactory i.e. ≥ 80 percent. * |
|  | b) The response rate is unsatisfactory, or the comparability between respondents and non-respondents is unsatisfactory. |
|  | c) No description of the response rate or the characteristics of the responders and the non-responders. |
| **4** | ***Comparability:*** The subjects in different outcome groups are comparable, based on the study design or analysis. Confounding factors are controlled **(Maximum 2 stars).** |
|  | a) The study controls for the most important factor (sex). * |
|  | b) Study controls for any additional factor (For example, residence, socio-economic status, education, occupation, stress, family type, physical activity, chronic diseases, etc.) * |
| **5** | ***Outcome: Ascertainment of outcome* (Maximum 2 stars).** |
|  | a) Validated measurement tool. ** |
|  | b) Non-validated measurement tool, but the tool is available or described. * |
|  | c) No description of the measurement tool. |
| **6** | ***Outcome:*** Statistical tests **(Maximum 1 star).** |
|  | a) The statistical test used to analyze the data is clearly described and appropriate, and the measurement of the association **(either multiple regression analysis OR odds ratio or relative risk or prevalence ratio)** is presented, including confidence intervals **OR** the probability level (p value).* |
|  | b) The statistical test is not appropriate, not described or incomplete. |

**Maximum score: 8*; and Minimum score: 0***
