# Supplementary material for: Prevalence of depression among the elderly (60 years and above) population in India, 1997–2016: a systematic review and meta-analysis
Source: BMC Public Health. 2019 Jun 27;19:832. doi: 10.1186/s12889-019-7136-z (PMC6598256; doi:10.1186/s12889-019-7136-z)

Supplementary figure 1: Estimated prevalence of depression among elderly persons in India- pooling included studies, 1997-2016 (Rural vs urban - subgroup analysis)

Study	ES (95% CI)	% Weight
Rural		
Abhishekh HA et al, 2013	14.3 (7.9, 24.3)	1.73
Arumugam B et al, 2013 (A)	79.5 (69.6, 86.8)	1.75
Arvind P et al, 2004	24.7 (19.9, 30.3)	1.83
Barua A et al, 2010	21.7 (18.6, 25.1)	1.86
Bayapa reddy PM et al, 2014	47.0 (43.6, 50.5)	1.86
Behera P et al, 2016	27.3 (23.2, 31.9)	1.85
Bodhare TN et al, 2013	44.7 (37.8, 51.8)	1.82
Dasgupta A et al, 2013	58.8 (48.2, 68.7)	1.75
Deshpande SS et al, 2011	41.1 (34.2, 48.4)	1.82
Ganguli M et al et al, 1999	46.4 (43.8, 49.1)	1.87
Goyal A et al, 2014 (A)	74.6 (62.2, 83.9)	1.70
Ishikawa M et al, 2016	7.9 (4.2, 14.3)	1.78
Jonas Jost B et al, 2014	58.5 (55.9, 61.1)	1.87
Kamble SV et al, 2009	31.4 (27.4, 35.6)	1.85
Kumar S et al, 2013	47.0 (42.2, 51.9)	1.85
Maulik S et al, 2012	53.7 (42.9, 64.0)	1.75
Nandi PS et al, 1997	55.2 (48.0, 62.2)	1.82
Patil SD et al, 2015	29.4 (25.1, 34.1)	1.85
Pilania M et al, 2016	14.4 (11.6, 17.7)	1.86
Pongiya UD et al, 2011	22.0 (14.7, 31.5)	1.76
Radhakrishnan S et al, 2013	58.7 (53.9, 63.5)	1.85
Sandhya GI et al, 2010	25.4 (20.4, 31.1)	1.83
Sengupta P et al, 2015 (A)	7.3 (6.0, 8.9)	1.87
Sharma K et al, 2016 (A)	7.3 (5.1, 10.2)	1.85
Sinha SP et al, 2013	42.7 (33.6, 52.4)	1.77
Suganathan S et al, 2016	70.4 (66.1, 74.5)	1.85
Sundru M et al, 2013 (A)	36.0 (32.3, 39.9)	1.86
Swarnalatha N et al, 2013	47.0 (42.2, 51.9)	1.85
Subtotal (I^2 = 98.7%, p = 0.0)	37.8 (29.9, 45.9)	50.98
Urban		
Ahmed MS et al, 2016	36.7 (33.5, 40.0)	1.86
Arumugam B et al, 2013 (B)	80.0 (70.6, 87.0)	1.76
D'souza L et al, 2015	51.9 (45.2, 58.6)	1.83
Dasgupta A et al, 2014	46.9 (38.6, 55.5)	1.79
Dhar G et al, 2013	59.8 (53.0, 66.3)	1.82
Dhuria M et al, 2014	45.6 (39.5, 51.8)	1.83
Dumbray SS et al, 2014	30.0 (21.9, 39.6)	1.77
Goel PK et al, 2014	9.4 (6.9, 12.7)	1.85
Goyal A et al, 2014 (B)	80.5 (66.0, 89.8)	1.64
Gupta A et al, 2015	15.6 (10.5, 22.5)	1.80
Gupta SK et al, 2012	9.6 (6.3, 14.4)	1.82
Jain RK et al, 2007	45.9 (39.1, 52.9)	1.82
Jariwala V et al, 2010	35.7 (25.5, 47.4)	1.73
Nair SS et al, 2013	32.4 (26.0, 39.5)	1.82
Payghan BS et al, 2013	41.7 (35.3, 48.3)	1.83
Poongothai S et al, 2009	28.5 (26.6, 30.4)	1.87
Pracheth R et al, 2013	29.4 (23.7, 35.7)	1.83
Raul A et al, 2013	21.3 (16.4, 27.2)	1.83
Saikia AM et al, 2016	17.3 (13.9, 21.3)	1.85
Sanjay TV et al, 2014	36.0 (27.3, 45.8)	1.77
Santosh A et al, 2014	33.3 (26.3, 41.2)	1.80
Seby K et al, 2011	19.3 (14.5, 25.3)	1.82
Sengupta P et al, 2015 (B)	10.1 (8.7, 11.5)	1.87
Sharma K et al, 2016 (B)	11.8 (9.0, 15.3)	1.85
Sundru M et al, 2013 (B)	27.3 (23.9, 31.0)	1.86
Thirthahalli C et al, 2014	37.8 (33.6, 42.3)	1.85
Yadav SP et al, 2013	15.9 (12.0, 20.8)	1.84
Subtotal (I^2 = 97.6%, p = 0.0)	32.1 (26.1, 38.5)	49.02
Heterogeneity between groups: p = 0.278		
Overall (I^2 = 98.5%, p = 0.0);	35.0 (29.9, 40.3)	100.00

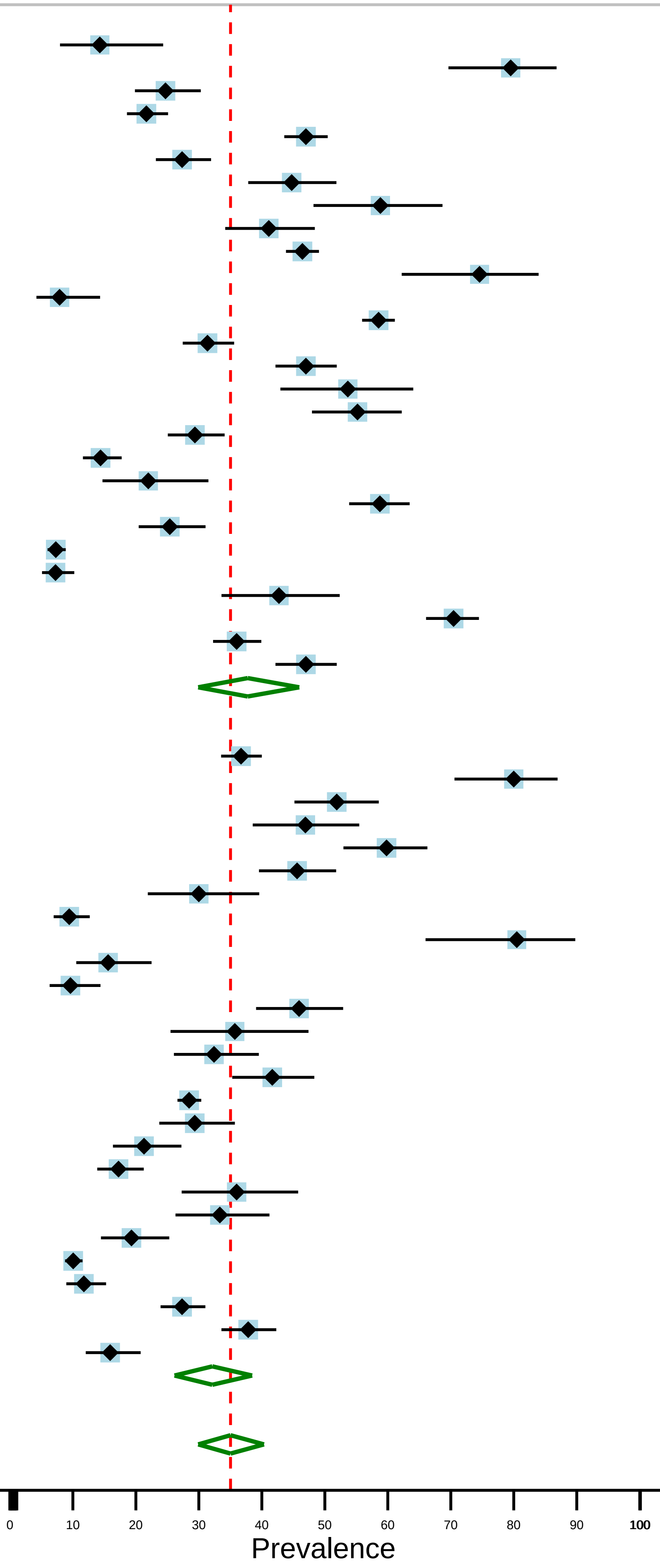

Supplementary figure 2: Estimated prevalence of depression among elderly persons in India-pooling included studies, 1997-2016 (Sampling techniques - subgroup analysis)

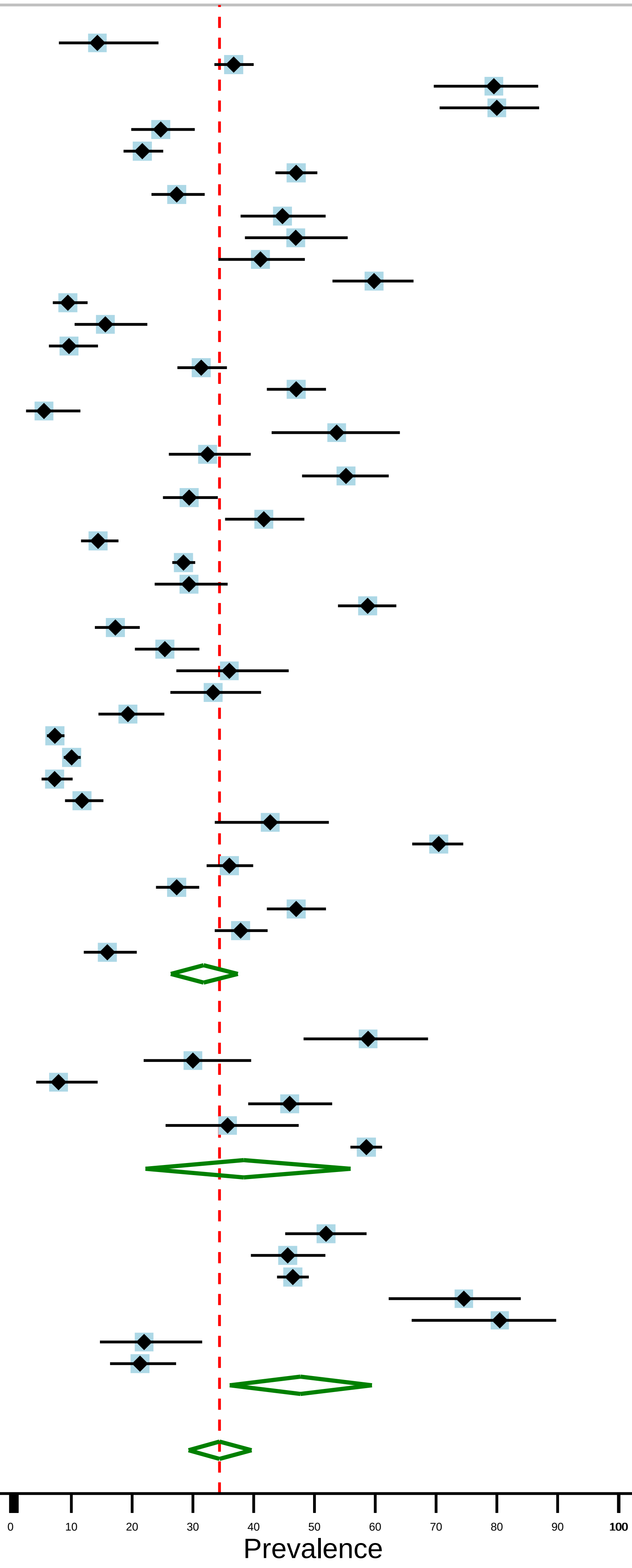

Study	ES (95% CI)	% Weight
Probability sampling		
Abhishekh HA et al, 2013	14.3 (7.9, 24.3)	1.70
Ahmed MS et al, 2016	36.7 (33.5, 40.0)	1.83
Arumugam B et al, 2013 (A)	79.5 (69.6, 86.8)	1.72
Arumugam B et al, 2013 (B)	80.0 (70.6, 87.0)	1.73
Arvind P et al, 2004	24.7 (19.9, 30.3)	1.80
Barua A et al, 2010	21.7 (18.6, 25.1)	1.83
Bayapa reddy PM et al, 2014	47.0 (43.6, 50.5)	1.83
Behera P et al, 2016	27.3 (23.2, 31.9)	1.82
Bodhare TN et al, 2013	44.7 (37.8, 51.8)	1.79
Dasgupta A et al, 2014	46.9 (38.6, 55.5)	1.76
Deshpande SS et al, 2011	41.1 (34.2, 48.4)	1.78
Dhar G et al, 2013	59.8 (53.0, 66.3)	1.79
Goel PK et al, 2014	9.4 (6.9, 12.7)	1.82
Gupta A et al, 2015	15.6 (10.5, 22.5)	1.77
Gupta SK et al, 2012	9.6 (6.3, 14.4)	1.79
Kamble SV et al, 2009	31.4 (27.4, 35.6)	1.82
Kumar S et al, 2013	47.0 (42.2, 51.9)	1.82
Mathias K et al	5.5 (2.5, 11.5)	1.75
Maulik S et al, 2012	53.7 (42.9, 64.0)	1.72
Nair SS et al, 2013	32.4 (26.0, 39.5)	1.79
Nandi PS et al, 1997	55.2 (48.0, 62.2)	1.79
Patil SD et al, 2015	29.4 (25.1, 34.1)	1.82
Payghan BS et al, 2013	41.7 (35.3, 48.3)	1.79
Pilania M et al, 2016	14.4 (11.6, 17.7)	1.82
Poongothai S et al, 2009	28.5 (26.6, 30.4)	1.84
Pracheth R et al, 2013	29.4 (23.7, 35.7)	1.79
Radhakrishnan S et al, 2013	58.7 (53.9, 63.5)	1.82
Saikia AM et al, 2016	17.3 (13.9, 21.3)	1.82
Sandhya GI et al, 2010	25.4 (20.4, 31.1)	1.80
Sanjay TV et al, 2014	36.0 (27.3, 45.8)	1.74
Santosh A et al, 2014	33.3 (26.3, 41.2)	1.77
Seby K et al, 2011	19.3 (14.5, 25.3)	1.79
Sengupta P et al, 2015 (A)	7.3 (6.0, 8.9)	1.84
Sengupta P et al, 2015 (B)	10.1 (8.7, 11.5)	1.84
Sharma K et al, 2016 (A)	7.3 (5.1, 10.2)	1.82
Sharma K et al, 2016 (B)	11.8 (9.0, 15.3)	1.82
Sinha SP et al, 2013	42.7 (33.6, 52.4)	1.74
Suganathan S et al, 2016	70.4 (66.1, 74.5)	1.82
Sundru M et al, 2013 (A)	36.0 (32.3, 39.9)	1.83
Sundru M et al, 2013 (B)	27.3 (23.9, 31.0)	1.83
Swarnalatha N et al, 2013	47.0 (42.2, 51.9)	1.82
Thirthahalli C et al, 2014	37.8 (33.6, 42.3)	1.82
Yadav SP et al, 2013	15.9 (12.0, 20.8)	1.80
Subtotal (I^2 = 98.4%, p = 0.0)	31.8 (26.4, 37.4)	77.22
Non-probability sampling		
Dasgupta A et al, 2013	58.8 (48.2, 68.7)	1.72
Dumbray SS et al, 2014	30.0 (21.9, 39.6)	1.74
Ishikawa M et al, 2016	7.9 (4.2, 14.3)	1.75
Jain RK et al, 2007	45.9 (39.1, 52.9)	1.79
Jariwala V et al, 2010	35.7 (25.5, 47.4)	1.70
Jonas Jost B et al, 2014	58.5 (55.9, 61.1)	1.84
Subtotal (I^2 = 97.2%, p = 0.0)	38.4 (22.2, 55.9)	10.54
Sampling methods not known		
D'souza L et al, 2015	51.9 (45.2, 58.6)	1.79
Dhuria M et al, 2014	45.6 (39.5, 51.8)	1.80
Ganguli M et al et al, 1999	46.4 (43.8, 49.1)	1.84
Goyal A et al, 2014 (A)	74.6 (62.2, 83.9)	1.68
Goyal A et al, 2014 (B)	80.5 (66.0, 89.8)	1.61
Pongiya UD et al, 2011	22.0 (14.7, 31.5)	1.73
Raul A et al, 2013	21.3 (16.4, 27.2)	1.79
Subtotal (I^2 = 95.3%, p = 0.0)	47.7 (36.1, 59.4)	12.24
Heterogeneity between groups: p = 0.048		
Overall (I^2 = 98.5%, p = 0.0);	34.4 (29.3, 39.6)	100.00

Supplementary figure 3: Estimated prevalence of depression among elderly persons in India- pooling included studies, 1997-2016 (Study instruments - subgroup analysis)

Study	ES (95% CI)	% Weight
CES-D		
Jonas Jost B et al, 2014	58.5 (55.9, 61.1)	1.84
Pongiya UD et al, 2011	22.0 (14.7, 31.5)	1.73
Thirthahalli C et al, 2014	37.8 (33.6, 42.3)	1.82
Subtotal (I^2 = .%, p = .)	39.5 (21.7, 58.9)	5.39
GDS		
Ahmed MS et al, 2016	36.7 (33.5, 40.0)	1.83
Arumugam B et al, 2013 (A)	79.5 (69.6, 86.8)	1.72
Arumugam B et al, 2013 (B)	80.0 (70.6, 87.0)	1.73
Arvind P et al, 2004	24.7 (19.9, 30.3)	1.80
Bayapa reddy PM et al, 2014	47.0 (43.6, 50.5)	1.83
Behera P et al, 2016	27.3 (23.2, 31.9)	1.82
D'souza L et al, 2015	51.9 (45.2, 58.6)	1.79
Dasgupta A et al, 2013	58.8 (48.2, 68.7)	1.72
Dasgupta A et al, 2014	46.9 (38.6, 55.5)	1.76
Deshpande SS et al, 2011	41.1 (34.2, 48.4)	1.78
Dhar G et al, 2013	59.8 (53.0, 66.3)	1.79
Dhuria M et al, 2014	45.6 (39.5, 51.8)	1.80
Dumbray SS et al, 2014	30.0 (21.9, 39.6)	1.74
Ganguli M et al et al, 1999	46.4 (43.8, 49.1)	1.84
Goel PK et al, 2014	9.4 (6.9, 12.7)	1.82
Goyal A et al, 2014 (A)	74.6 (62.2, 83.9)	1.68
Goyal A et al, 2014 (B)	80.5 (66.0, 89.8)	1.61
Gupta A et al, 2015	15.6 (10.5, 22.5)	1.77
Gupta SK et al, 2012	9.6 (6.3, 14.4)	1.79
Jain RK et al, 2007	45.9 (39.1, 52.9)	1.79
Kumar S et al, 2013	47.0 (42.2, 51.9)	1.82
Maulik S et al, 2012	53.7 (42.9, 64.0)	1.72
Nair SS et al, 2013	32.4 (26.0, 39.5)	1.79
Patil SD et al, 2015	29.4 (25.1, 34.1)	1.82
Payghan BS et al, 2013	41.7 (35.3, 48.3)	1.79
Pilania M et al, 2016	14.4 (11.6, 17.7)	1.82
Pracheth R et al, 2013	29.4 (23.7, 35.7)	1.79
Radhakrishnan S et al, 2013	58.7 (53.9, 63.5)	1.82
Saikia AM et al, 2016	17.3 (13.9, 21.3)	1.82
Sandhya GI et al, 2010	25.4 (20.4, 31.1)	1.80
Sanjay TV et al, 2014	36.0 (27.3, 45.8)	1.74
Santosh A et al, 2014	33.3 (26.3, 41.2)	1.77
Seby K et al, 2011	19.3 (14.5, 25.3)	1.79
Sengupta P et al, 2015 (A)	7.3 (6.0, 8.9)	1.84
Sengupta P et al, 2015 (B)	10.1 (8.7, 11.5)	1.84
Sinha SP et al, 2013	42.7 (33.6, 52.4)	1.74
Suganathan S et al, 2016	70.4 (66.1, 74.5)	1.82
Sundru M et al, 2013 (A)	36.0 (32.3, 39.9)	1.83
Sundru M et al, 2013 (B)	27.3 (23.9, 31.0)	1.83
Swarnalatha N et al, 2013	47.0 (42.2, 51.9)	1.82
Yadav SP et al, 2013	15.9 (12.0, 20.8)	1.80
Subtotal (I^2 = 98.5%, p = 0.0)	37.9 (31.5, 44.5)	73.22
HDRS		
Abhishekh HA et al, 2013	14.3 (7.9, 24.3)	1.70
Sharma K et al, 2016 (A)	7.3 (5.1, 10.2)	1.82
Sharma K et al, 2016 (B)	11.8 (9.0, 15.3)	1.82
Subtotal (I^2 = .%, p = .)	10.2 (6.5, 14.6)	5.33
PHQ		
Bodhare TN et al, 2013	44.7 (37.8, 51.8)	1.79
Ishikawa M et al, 2016	7.9 (4.2, 14.3)	1.75
Mathias K et al	5.5 (2.5, 11.5)	1.75
Poongothai S et al, 2009	28.5 (26.6, 30.4)	1.84
Subtotal (I^2 = 96.9%, p = 0.0)	19.7 (7.5, 35.7)	7.13
Others		
Barua A et al, 2010	21.7 (18.6, 25.1)	1.83
Jariwala V et al, 2010	35.7 (25.5, 47.4)	1.70
Kamble SV et al, 2009	31.4 (27.4, 35.6)	1.82
Nandi PS et al, 1997	55.2 (48.0, 62.2)	1.79
Raul A et al, 2013	21.3 (16.4, 27.2)	1.79
Subtotal (I^2 = 95.0%, p = 0.0)	32.3 (21.8, 43.8)	8.93
Heterogeneity between groups: p = 0.000		
Overall (I^2 = 98.5%, p = 0.0);	34.4 (29.3, 39.6)	100.00

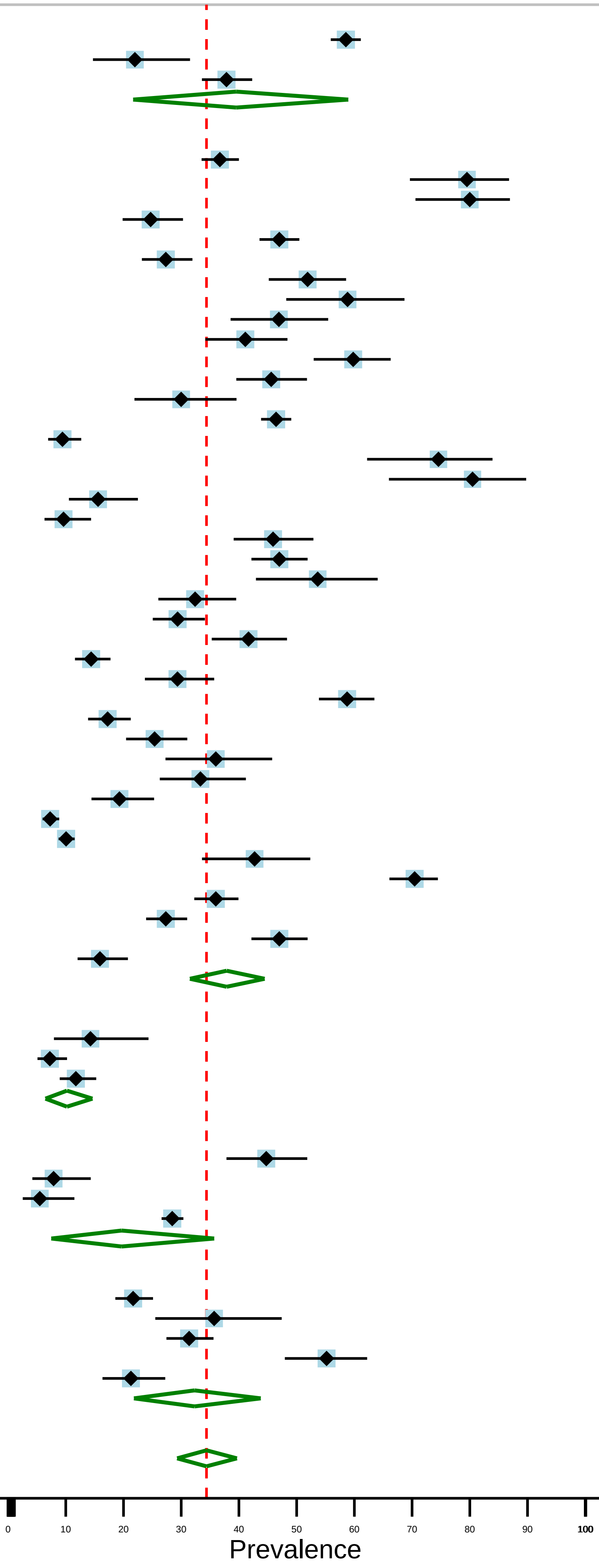

Supplementary figure 4: Estimated prevalence of depression among elderly persons in India- pooling included studies, 1997-2016 (Study instruments for geriatric vs. nongeriatric age groups - subgroup analysis)

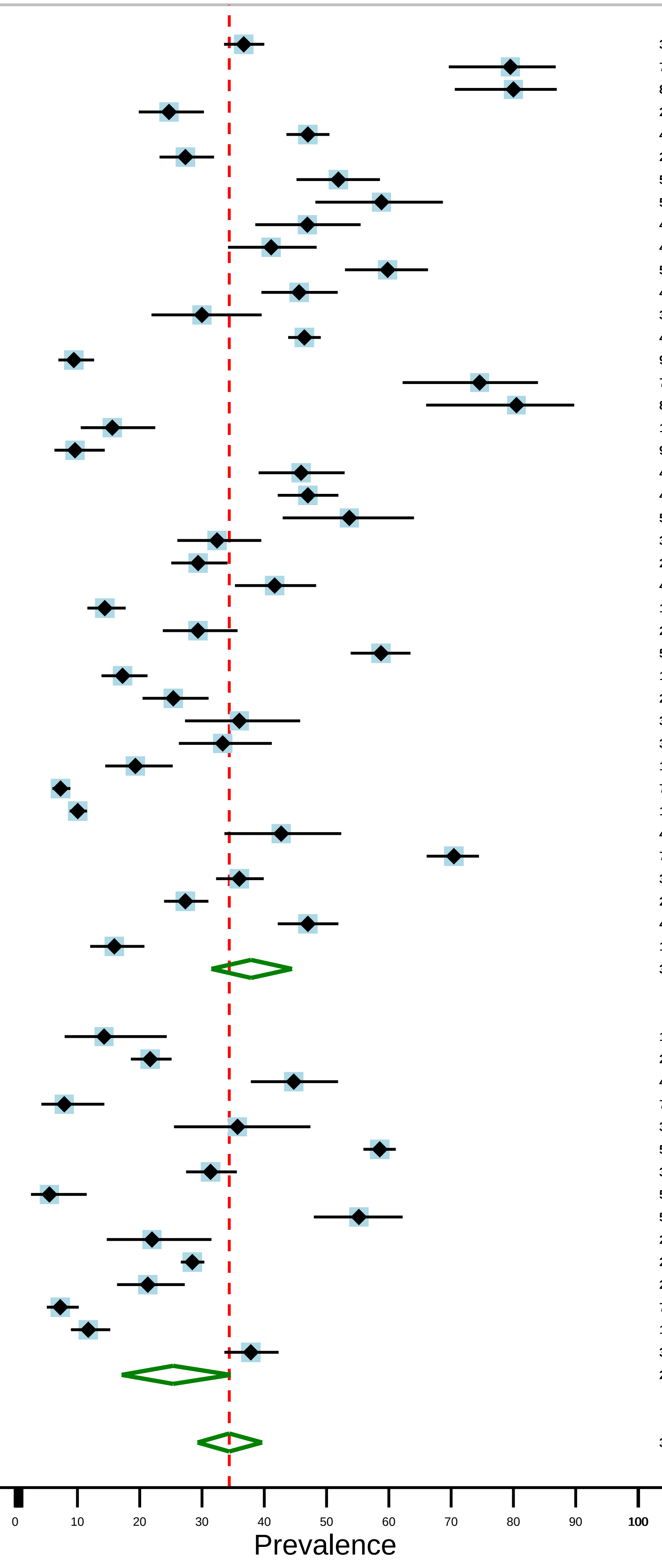

Study	ES (95% CI)	% Weight
GDS		
Ahmed MS et al, 2016	36.7 (33.5, 40.0)	1.83
Arumugam B et al, 2013 (A)	79.5 (69.6, 86.8)	1.72
Arumugam B et al, 2013 (B)	80.0 (70.6, 87.0)	1.73
Arvind P et al, 2004	24.7 (19.9, 30.3)	1.80
Bayapa reddy PM et al, 2014	47.0 (43.6, 50.5)	1.83
Behera P et al, 2016	27.3 (23.2, 31.9)	1.82
D'souza L et al, 2015	51.9 (45.2, 58.6)	1.79
Dasgupta A et al, 2013	58.8 (48.2, 68.7)	1.72
Dasgupta A et al, 2014	46.9 (38.6, 55.5)	1.76
Deshpande SS et al, 2011	41.1 (34.2, 48.4)	1.78
Dhar G et al, 2013	59.8 (53.0, 66.3)	1.79
Dhuria M et al, 2014	45.6 (39.5, 51.8)	1.80
Dumbray SS et al, 2014	30.0 (21.9, 39.6)	1.74
Ganguli M et al et al, 1999	46.4 (43.8, 49.1)	1.84
Goel PK et al, 2014	9.4 (6.9, 12.7)	1.82
Goyal A et al, 2014 (A)	74.6 (62.2, 83.9)	1.68
Goyal A et al, 2014 (B)	80.5 (66.0, 89.8)	1.61
Gupta A et al, 2015	15.6 (10.5, 22.5)	1.77
Gupta SK et al, 2012	9.6 (6.3, 14.4)	1.79
Jain RK et al, 2007	45.9 (39.1, 52.9)	1.79
Kumar S et al, 2013	47.0 (42.2, 51.9)	1.82
Maulik S et al, 2012	53.7 (42.9, 64.0)	1.72
Nair SS et al, 2013	32.4 (26.0, 39.5)	1.79
Patil SD et al, 2015	29.4 (25.1, 34.1)	1.82
Payghan BS et al, 2013	41.7 (35.3, 48.3)	1.79
Pilania M et al, 2016	14.4 (11.6, 17.7)	1.82
Pracheth R et al, 2013	29.4 (23.7, 35.7)	1.79
Radhakrishnan S et al, 2013	58.7 (53.9, 63.5)	1.82
Saikia AM et al, 2016	17.3 (13.9, 21.3)	1.82
Sandhya GI et al, 2010	25.4 (20.4, 31.1)	1.80
Sanjay TV et al, 2014	36.0 (27.3, 45.8)	1.74
Santosh A et al, 2014	33.3 (26.3, 41.2)	1.77
Seby K et al, 2011	19.3 (14.5, 25.3)	1.79
Sengupta P et al, 2015 (A)	7.3 (6.0, 8.9)	1.84
Sengupta P et al, 2015 (B)	10.1 (8.7, 11.5)	1.84
Sinha SP et al, 2013	42.7 (33.6, 52.4)	1.74
Suganathan S et al, 2016	70.4 (66.1, 74.5)	1.82
Sundru M et al, 2013 (A)	36.0 (32.3, 39.9)	1.83
Sundru M et al, 2013 (B)	27.3 (23.9, 31.0)	1.83
Swarnalatha N et al, 2013	47.0 (42.2, 51.9)	1.82
Yadav SP et al, 2013	15.9 (12.0, 20.8)	1.80
Subtotal (I^2 = 98.5%, p = 0.0)	37.9 (31.5, 44.5)	73.22
Others		
Abhishekh HA et al, 2013	14.3 (7.9, 24.3)	1.70
Barua A et al, 2010	21.7 (18.6, 25.1)	1.83
Bodhare TN et al, 2013	44.7 (37.8, 51.8)	1.79
Ishikawa M et al, 2016	7.9 (4.2, 14.3)	1.75
Jariwala V et al, 2010	35.7 (25.5, 47.4)	1.70
Jonas Jost B et al, 2014	58.5 (55.9, 61.1)	1.84
Kamble SV et al, 2009	31.4 (27.4, 35.6)	1.82
Mathias K et al	5.5 (2.5, 11.5)	1.75
Nandi PS et al, 1997	55.2 (48.0, 62.2)	1.79
Pongiya UD et al, 2011	22.0 (14.7, 31.5)	1.73
Poongothai S et al, 2009	28.5 (26.6, 30.4)	1.84
Raul A et al, 2013	21.3 (16.4, 27.2)	1.79
Sharma K et al, 2016 (A)	7.3 (5.1, 10.2)	1.82
Sharma K et al, 2016 (B)	11.8 (9.0, 15.3)	1.82
Thirthahalli C et al, 2014	37.8 (33.6, 42.3)	1.82
Subtotal (I^2 = 98.5%, p = 0.0)	25.4 (17.1, 34.6)	26.78
Heterogeneity between groups: p = 0.029		
Overall (I^2 = 98.5%, p = 0.0);	34.4 (29.3, 39.6)	100.00

Supplementary figure 5: Estimated prevalence of depression among elderly persons in India- pooling included studies, 1997-2016 (EAG vs Non-EAG states of India - subgroup analysis)

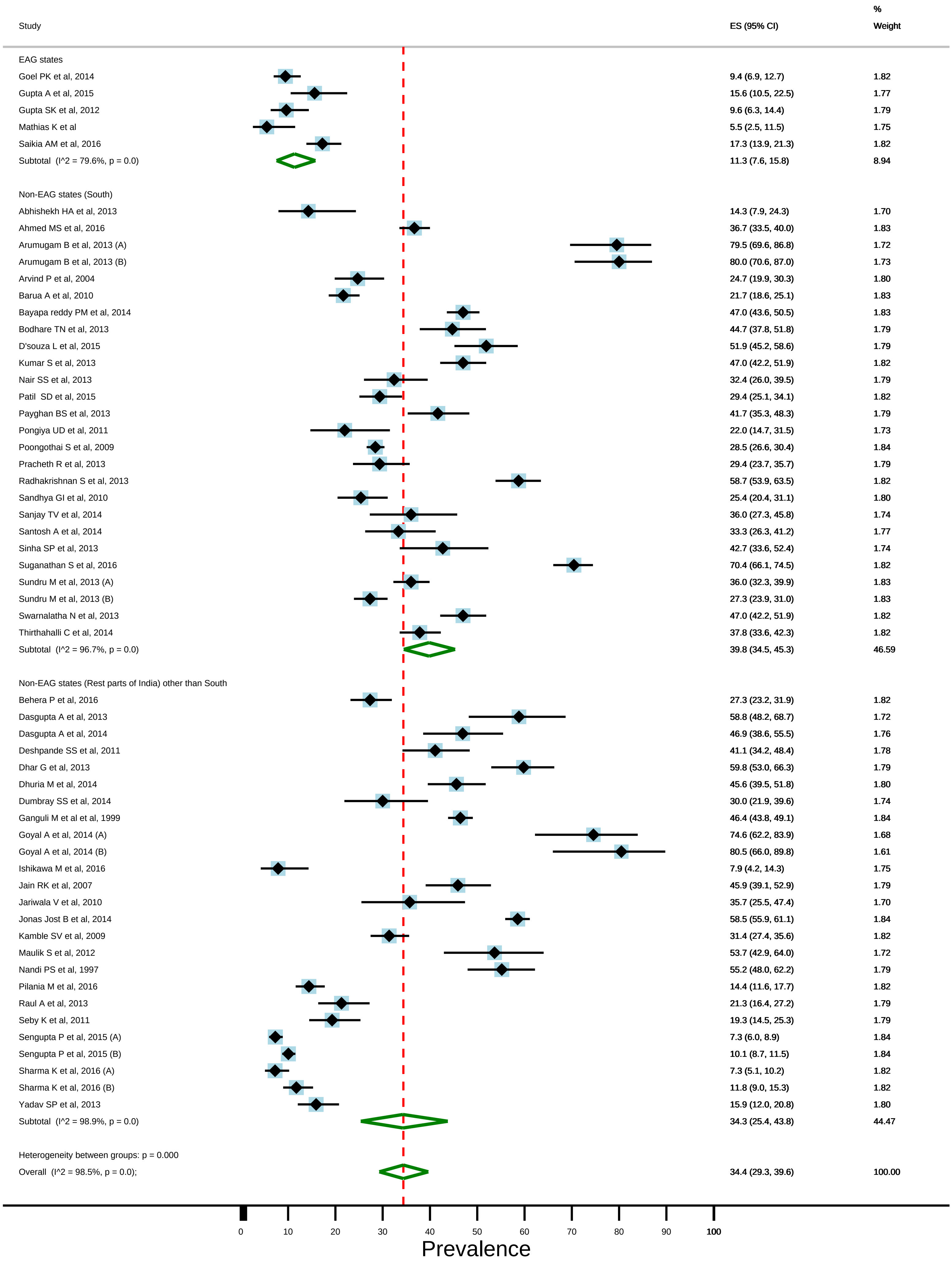

Supplementary figure 6: Estimated prevalence of depression among elderly persons in India- pooling included studies, 1997-2016 (Geographical regions of India- subgroup analysis)

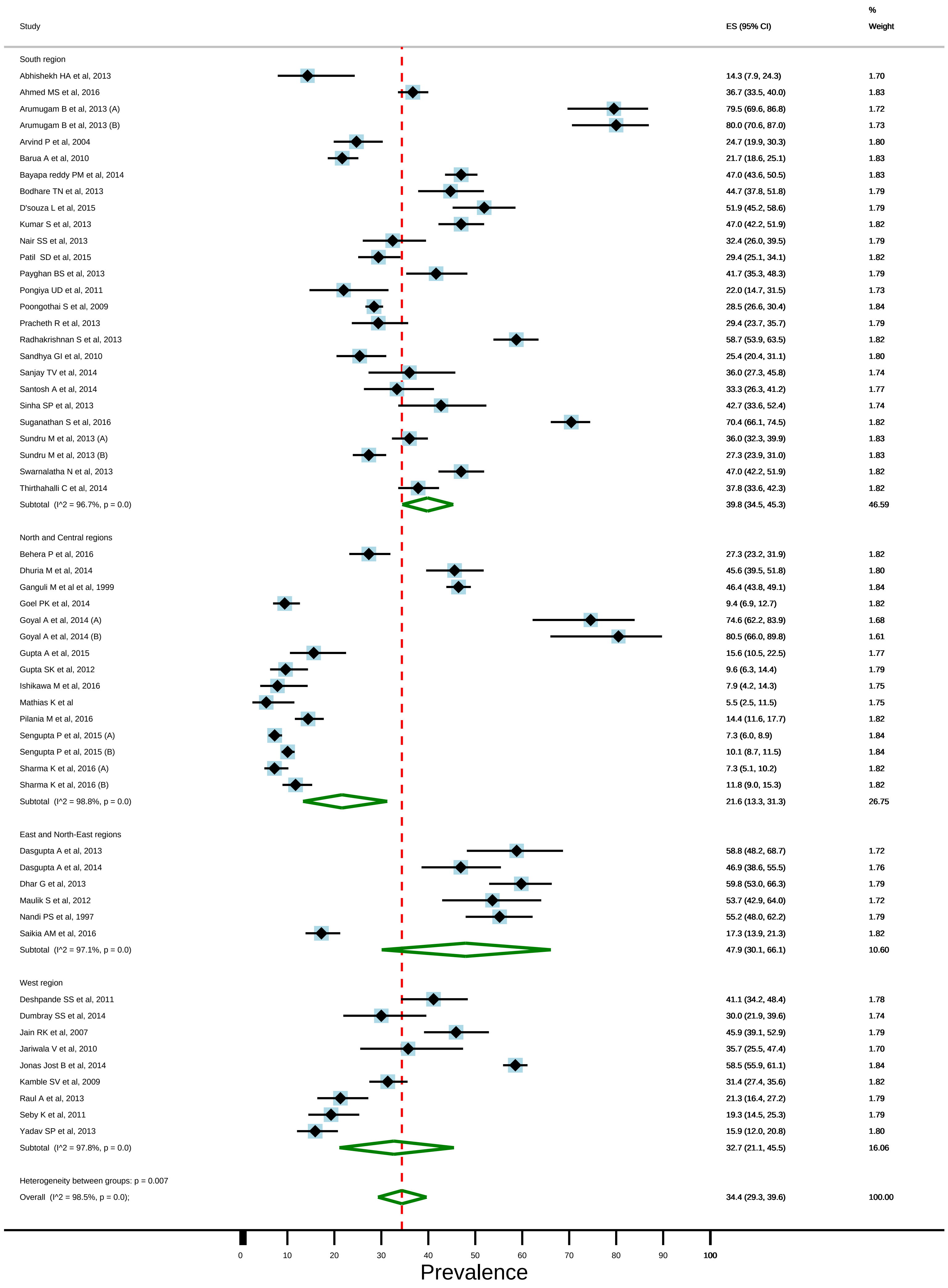

Supplementary figure 7: Estimated prevalence of depression among elderly persons in India-pooling included studies, 1997-2016 (Time period (decades) - subgroup analysis)

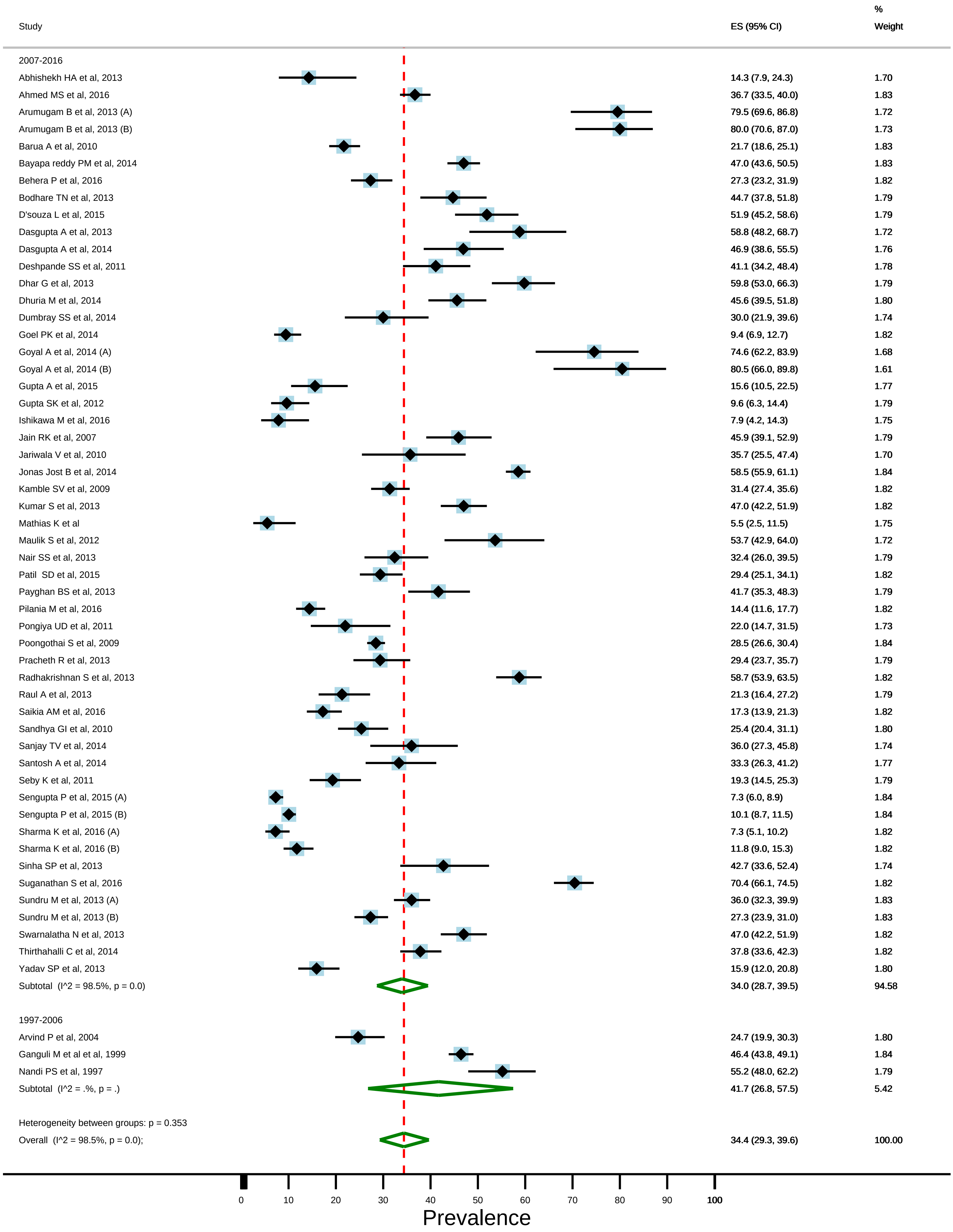

Supplementary figure 8: Estimated prevalence of depression among elderly persons in India- pooling included studies,1997-2016 (Studies with inclusion age> 60 years only - sensitivity analysis)

Study	ES (95% CI)	% Weight
Abhishekh HA et al, 2013	14.3 (7.9, 24.3)	1.76
Ahmed MS et al, 2016	36.7 (33.5, 40.0)	1.90
Arumugam B et al, 2013 (A)	79.5 (69.6, 86.8)	1.79
Arumugam B et al, 2013 (B)	80.0 (70.6, 87.0)	1.79
Arvind P et al, 2004	24.7 (19.9, 30.3)	1.87
Barua A et al, 2010	21.7 (18.6, 25.1)	1.89
Bayapa reddy PM et al, 2014	47.0 (43.6, 50.5)	1.90
Behera P et al, 2016	27.3 (23.2, 31.9)	1.88
Bodhare TN et al, 2013	44.7 (37.8, 51.8)	1.85
D'souza L et al, 2015	51.9 (45.2, 58.6)	1.86
Dasgupta A et al, 2013	58.8 (48.2, 68.7)	1.79
Dasgupta A et al, 2014	46.9 (38.6, 55.5)	1.83
Dhar G et al, 2013	59.8 (53.0, 66.3)	1.86
Dhuria M et al, 2014	45.6 (39.5, 51.8)	1.87
Dumbray SS et al, 2014	30.0 (21.9, 39.6)	1.81
Ganguli M et al et al, 1999	46.4 (43.8, 49.1)	1.90
Goel PK et al, 2014	9.4 (6.9, 12.7)	1.88
Goyal A et al, 2014 (A)	74.6 (62.2, 83.9)	1.74
Goyal A et al, 2014 (B)	80.5 (66.0, 89.8)	1.67
Gupta A et al, 2015	15.6 (10.5, 22.5)	1.84
Gupta SK et al, 2012	9.6 (6.3, 14.4)	1.86
Ishikawa M et al, 2016	7.9 (4.2, 14.3)	1.82
Jain RK et al, 2007	45.9 (39.1, 52.9)	1.86
Jariwala V et al, 2010	35.7 (25.5, 47.4)	1.76
Jonas Jost B et al, 2014	58.5 (55.9, 61.1)	1.90
Kamble SV et al, 2009	31.4 (27.4, 35.6)	1.89
Kumar S et al, 2013	47.0 (42.2, 51.9)	1.88
Mathias K et al	5.5 (2.5, 11.5)	1.81
Maulik S et al, 2012	53.7 (42.9, 64.0)	1.78
Nair SS et al, 2013	32.4 (26.0, 39.5)	1.85
Nandi PS et al, 1997	55.2 (48.0, 62.2)	1.85
Patil SD et al, 2015	29.4 (25.1, 34.1)	1.88
Payghan BS et al, 2013	41.7 (35.3, 48.3)	1.86
Pilania M et al, 2016	14.4 (11.6, 17.7)	1.89
Pongiya UD et al, 2011	22.0 (14.7, 31.5)	1.80
Poongothai S et al, 2009	28.5 (26.6, 30.4)	1.91
Pracheth R et al, 2013	29.4 (23.7, 35.7)	1.86
Radhakrishnan S et al, 2013	58.7 (53.9, 63.5)	1.88
Raul A et al, 2013	21.3 (16.4, 27.2)	1.86
Saikia AM et al, 2016	17.3 (13.9, 21.3)	1.88
Sandhya GI et al, 2010	25.4 (20.4, 31.1)	1.87
Sanjay TV et al, 2014	36.0 (27.3, 45.8)	1.81
Santosh A et al, 2014	33.3 (26.3, 41.2)	1.84
Sengupta P et al, 2015 (A)	7.3 (6.0, 8.9)	1.90
Sengupta P et al, 2015 (B)	10.1 (8.7, 11.5)	1.91
Sharma K et al, 2016 (A)	7.3 (5.1, 10.2)	1.88
Sharma K et al, 2016 (B)	11.8 (9.0, 15.3)	1.88
Sinha SP et al, 2013	42.7 (33.6, 52.4)	1.81
Suganathan S et al, 2016	70.4 (66.1, 74.5)	1.89
Sundru M et al, 2013 (A)	36.0 (32.3, 39.9)	1.89
Sundru M et al, 2013 (B)	27.3 (23.9, 31.0)	1.89
Swarnalatha N et al, 2013	47.0 (42.2, 51.9)	1.88
Thirthahalli C et al, 2014	37.8 (33.6, 42.3)	1.89
Yadav SP et al, 2013	15.9 (12.0, 20.8)	1.87
Overall (I^2 = 98.5%, p = 0.0)	34.6 (29.3, 40.0)	100.00

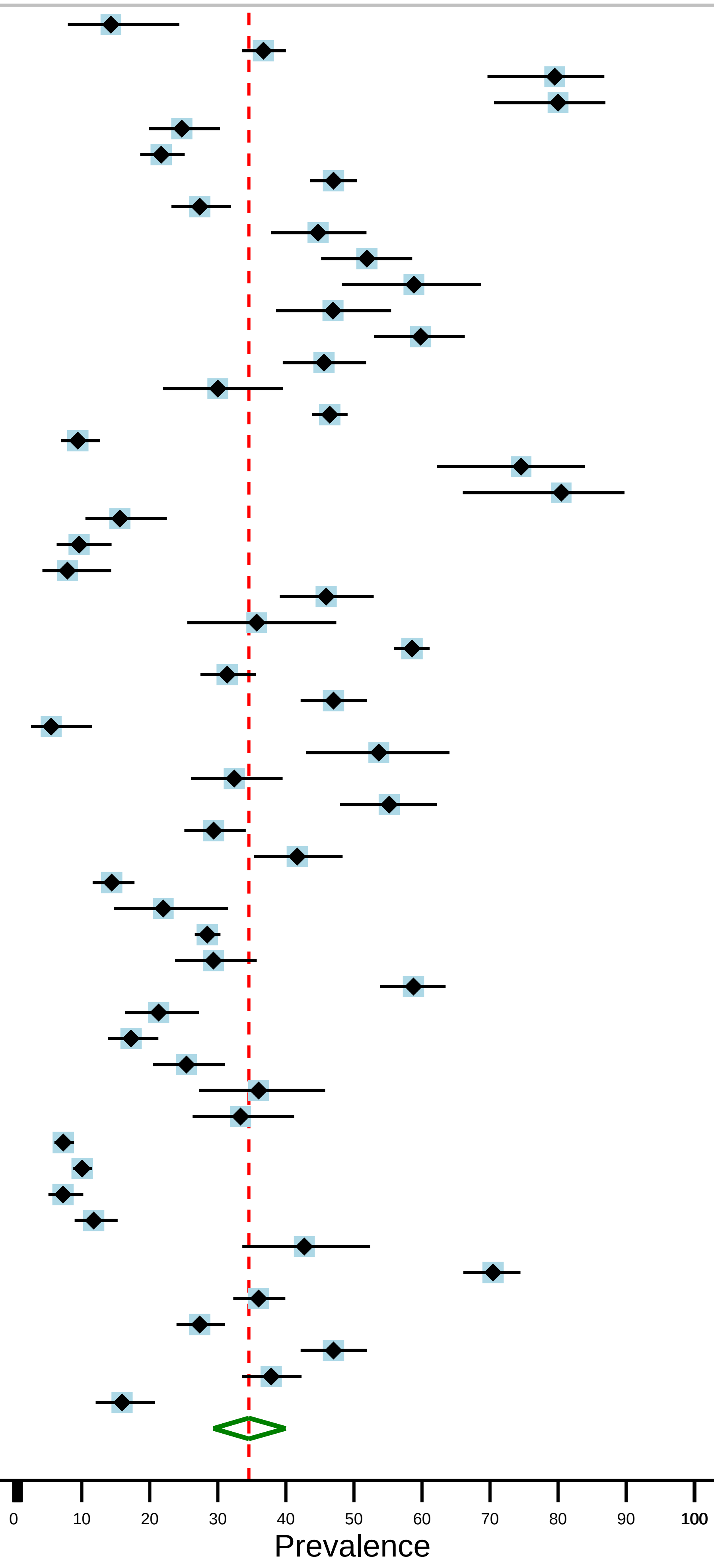

Supplementary figure 9: Estimated prevalence of depression among elderly persons in India- pooling included studies, 1997-2016 (Excluding studies with sample size <100 - sensitivity analysis)

Study	ES (95% CI)	% Weight
Ahmed MS et al, 2016	36.7 (33.5, 40.0)	2.26
Arvind P et al, 2004	24.7 (19.9, 30.3)	2.22
Barua A et al, 2010	21.7 (18.6, 25.1)	2.25
Bayapa reddy PM et al, 2014	47.0 (43.6, 50.5)	2.26
Behera P et al, 2016	27.3 (23.2, 31.9)	2.24
Bodhare TN et al, 2013	44.7 (37.8, 51.8)	2.20
D'souza L et al, 2015	51.9 (45.2, 58.6)	2.21
Dasgupta A et al, 2014	46.9 (38.6, 55.5)	2.17
Deshpande SS et al, 2011	41.1 (34.2, 48.4)	2.20
Dhar G et al, 2013	59.8 (53.0, 66.3)	2.21
Dhuria M et al, 2014	45.6 (39.5, 51.8)	2.22
Ganguli M et al et al, 1999	46.4 (43.8, 49.1)	2.26
Goel PK et al, 2014	9.4 (6.9, 12.7)	2.24
Gupta A et al, 2015	15.6 (10.5, 22.5)	2.18
Gupta SK et al, 2012	9.6 (6.3, 14.4)	2.21
Ishikawa M et al, 2016	7.9 (4.2, 14.3)	2.15
Jain RK et al, 2007	45.9 (39.1, 52.9)	2.20
Jonas Jost B et al, 2014	58.5 (55.9, 61.1)	2.26
Kamble SV et al, 2009	31.4 (27.4, 35.6)	2.24
Kumar S et al, 2013	47.0 (42.2, 51.9)	2.24
Mathias K et al	5.5 (2.5, 11.5)	2.15
Nair SS et al, 2013	32.4 (26.0, 39.5)	2.20
Nandi PS et al, 1997	55.2 (48.0, 62.2)	2.20
Patil SD et al, 2015	29.4 (25.1, 34.1)	2.24
Payghan BS et al, 2013	41.7 (35.3, 48.3)	2.21
Pilania M et al, 2016	14.4 (11.6, 17.7)	2.25
Poongothai S et al, 2009	28.5 (26.6, 30.4)	2.27
Pracheth R et al, 2013	29.4 (23.7, 35.7)	2.21
Radhakrishnan S et al, 2013	58.7 (53.9, 63.5)	2.24
Raul A et al, 2013	21.3 (16.4, 27.2)	2.21
Saikia AM et al, 2016	17.3 (13.9, 21.3)	2.24
Sandhya GI et al, 2010	25.4 (20.4, 31.1)	2.22
Santosh A et al, 2014	33.3 (26.3, 41.2)	2.18
Seby K et al, 2011	19.3 (14.5, 25.3)	2.20
Sengupta P et al, 2015 (A)	7.3 (6.0, 8.9)	2.26
Sengupta P et al, 2015 (B)	10.1 (8.7, 11.5)	2.27
Sharma K et al, 2016 (A)	7.3 (5.1, 10.2)	2.24
Sharma K et al, 2016 (B)	11.8 (9.0, 15.3)	2.24
Sinha SP et al, 2013	42.7 (33.6, 52.4)	2.14
Suganathan S et al, 2016	70.4 (66.1, 74.5)	2.24
Sundru M et al, 2013 (A)	36.0 (32.3, 39.9)	2.25
Sundru M et al, 2013 (B)	27.3 (23.9, 31.0)	2.25
Swarnalatha N et al, 2013	47.0 (42.2, 51.9)	2.24
Thirthahalli C et al, 2014	37.8 (33.6, 42.3)	2.24
Yadav SP et al, 2013	15.9 (12.0, 20.8)	2.22
Overall (I^2 = 98.6%, p = 0.0)	30.7 (25.4, 36.3)	100.00

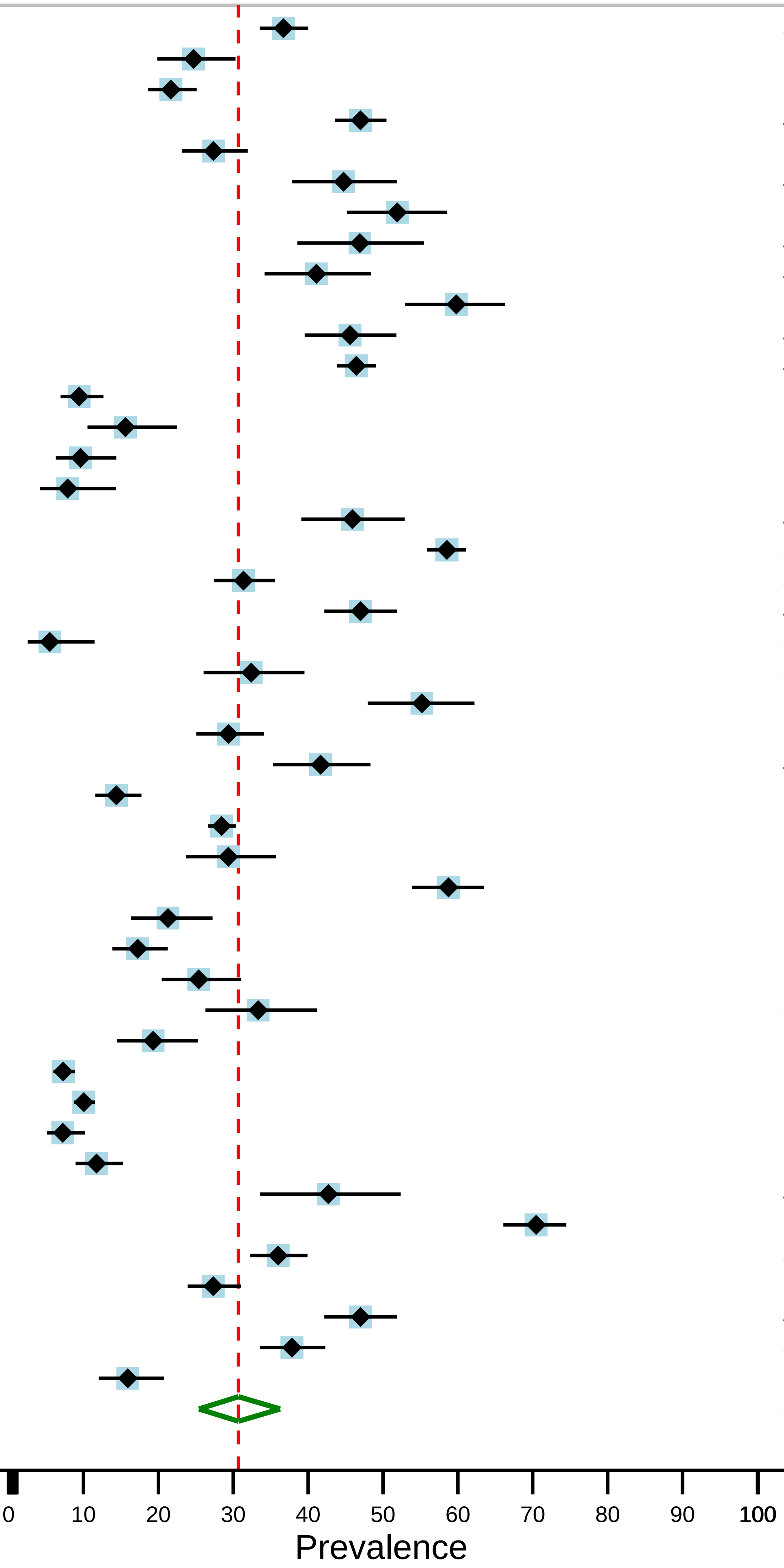

Supplementary figure 10: Estimated prevalence of depression among elderly persons in India- pooling included studies 1997-2016 (Studies with dementia exclusion - sensitivity analysis)

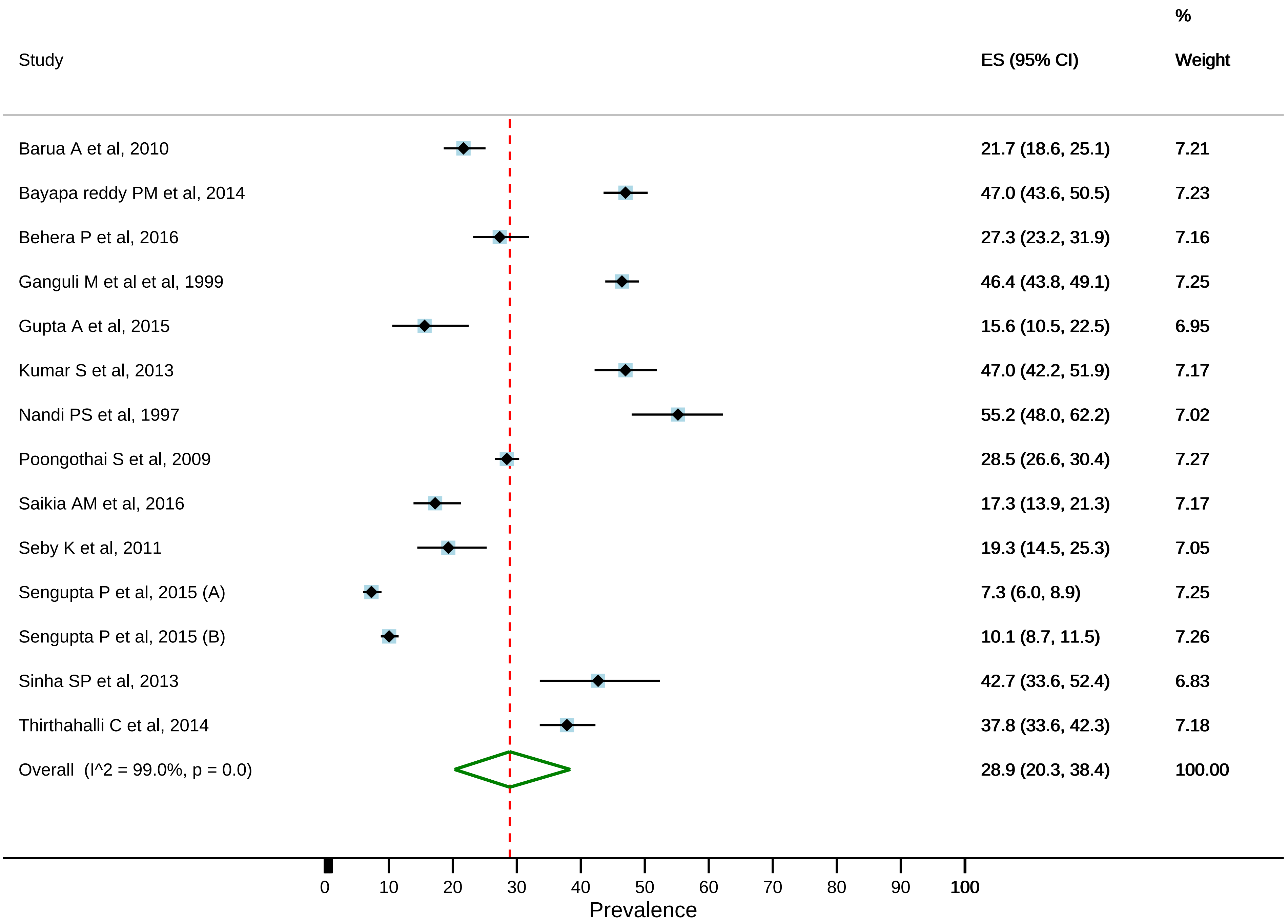

Supplement: Supplementary file 3 — Figure S1. Estimated prevalence of depression among elderly persons in India pooling included studies, 1997–2016 (Rural vs. urban – subgroup analysis). Figure S2. Estimated prevalence of depression among elderly persons in India pooling included studies, 1997–2016 (Sampling techniques – subgroup analysis). Figure S3. Estimated prevalence of depression among elderly persons in India pooling included studies, 1997–2016 (Study instruments – subgroup analysis). Figure S4. Estimated prevalence of depression among elderly persons in India pooling included studies, 1997–2016 (Study instrument for geriatric vs. nongeriatric age groups – subgroup analysis). Figure S5. Estimated prevalence of depression among elderly persons in India- pooling included studies: 1997–2016 (EAG vs Non-EAG state of India – subgroup analysis). Figure S6. Estimated prevalence of depression among elderly persons in India pooling included studies, 1997–2016 (Geographical regions of India – subgroup analysis). Figure S7. Estimated prevalence of depression among elderly persons in India pooling included studies, 1997–2016 (Time period – subgroup analysis). Figure S8. Estimated prevalence of depression among elderly persons in India pooling included studies, 1997–2016 (Studies with inclusion age > 60 years only – sensitivity analysis). Figure S9. Estimated prevalence of depression among elderly persons in India pooling included studies, 1997–2016 (Excluding studies with sample size < 100 – sensitivity analysis). Figure S10. Estimated prevalence of depression among elderly persons in India- pooling included studies 1997–2016 (Studies with dementia exclusion – sensitivity analysis). (PDF 237 kb) [file 12889_2019_7136_MOESM3_ESM.pdf]
